# Large stocks of peatland carbon and nitrogen are vulnerable to permafrost thaw

**DOI:** 10.1073/pnas.1916387117

**Published:** 2020-08-10

**Authors:** Gustaf Hugelius, Julie Loisel, Sarah Chadburn, Robert B. Jackson, Miriam Jones, Glen MacDonald, Maija Marushchak, David Olefeldt, Maara Packalen, Matthias B. Siewert, Claire Treat, Merritt Turetsky, Carolina Voigt, Zicheng Yu

**Affiliations:** ^a^Department of Physical Geography, Stockholm University, 106 91 Stockholm, Sweden;; ^b^Bolin Centre for Climate Research, Stockholm University, 106 91 Stockholm, Sweden;; ^c^Department of Earth System Science, Stanford University, Stanford, CA 94305;; ^d^Department of Geography, Texas A&M University, College Station, TX 77843;; ^e^Department of Mathematics, University of Exeter, Exeter EX4 4QE, United Kingdom;; ^f^Woods Institute for the Environment and Precourt Institute for Energy, Stanford University, Stanford, CA 94305;; ^g^Florence Bascom Geoscience Center, US Geological Survey, Reston, VA 20192;; ^h^Department of Geography, University of California, Los Angeles, CA 90095-1524;; ^i^Department of Biological and Environmental Science, University of Jyväskylä, FI-40014 Jyväskylä, Finland;; ^j^Department of Geography, University of Toronto, Toronto, ON M5S 3G3, Canada;; ^k^Department of Renewable Resources, University of Alberta, Edmonton, AB T6G 2R3, Canada;; ^l^Department of Ecology and Environmental Science, Umeå University, 907 36 Umeå, Sweden;; ^m^Earth Systems Research Center, Institute for the Study of Earth, Oceans, and Space, University of New Hampshire, Durham, NH 03824;; ^n^Department of Integrative Biology, University of Guelph, Guelph, ON N1G 2W1, Canada;; ^o^Institute of Arctic and Alpine Research, University of Colorado, Boulder, CO 80309;; ^p^Department of Geography, University of Montreal, Montreal, QC H2V 0B3, Canada;; ^q^Department of Earth and Environmental Sciences, Lehigh University, Bethlehem, PA 18015;; ^r^Institute for Peat and Mire Research, School of Geographical Sciences, Northeast Normal University, 130024 Changchun, China

**Keywords:** northern peatlands, carbon stocks, nitrogen stocks, greenhouse gas fluxes, permafrost thaw

## Abstract

Northern peatlands have accumulated large stocks of organic carbon (C) and nitrogen (N), but their spatial distribution and vulnerability to climate warming remain uncertain. Here, we used machine-learning techniques with extensive peat core data (*n* > 7,000) to create observation-based maps of northern peatland C and N stocks, and to assess their response to warming and permafrost thaw. We estimate that northern peatlands cover 3.7 ± 0.5 million km^2^ and store 415 ± 150 Pg C and 10 ± 7 Pg N. Nearly half of the peatland area and peat C stocks are permafrost affected. Using modeled global warming stabilization scenarios (from 1.5 to 6 °C warming), we project that the current sink of atmospheric C (0.10 ± 0.02 Pg C⋅y^−1^) in northern peatlands will shift to a C source as 0.8 to 1.9 million km^2^ of permafrost-affected peatlands thaw. The projected thaw would cause peatland greenhouse gas emissions equal to ∼1% of anthropogenic radiative forcing in this century. The main forcing is from methane emissions (0.7 to 3 Pg cumulative CH_4_-C) with smaller carbon dioxide forcing (1 to 2 Pg CO_2_-C) and minor nitrous oxide losses. We project that initial CO_2_-C losses reverse after ∼200 y, as warming strengthens peatland C-sinks. We project substantial, but highly uncertain, additional losses of peat into fluvial systems of 10 to 30 Pg C and 0.4 to 0.9 Pg N. The combined gaseous and fluvial peatland C loss estimated here adds 30 to 50% onto previous estimates of permafrost-thaw C losses, with southern permafrost regions being the most vulnerable.

## Significance

Over many millennia, northern peatlands have accumulated large amounts of carbon and nitrogen, thus cooling the global climate. Over shorter timescales, peatland disturbances can trigger losses of peat and release of greenhouses gases. Despite their importance to the global climate, peatlands remain poorly mapped, and the vulnerability of permafrost peatlands to warming is uncertain. This study compiles over 7,000 field observations to present a data-driven map of northern peatlands and their carbon and nitrogen stocks. We use these maps to model the impact of permafrost thaw on peatlands and find that warming will likely shift the greenhouse gas balance of northern peatlands. At present, peatlands cool the climate, but anthropogenic warming can shift them into a net source of warming.

Northern peatlands are an important and dynamic component of the climate system. They hold large stocks of organic C and N and have been a persistent long-term sink of atmospheric carbon dioxide (CO_2_), but are a source of methane (CH_4_) (1–4). Climate warming and increased atmospheric CO_2_ are projected to generally increase northern peat C sequestration as increases in vegetation productivity exceed increases in decomposition (5–7). However, it remains unclear to what degree this increased peat growth may be offset by climate-driven increases in peatland disturbances, including higher fire frequency (8), drought (9), and thawing of permafrost (10). There is field-based evidence of both net losses and net gains of peat resulting from thaw of permafrost peatlands, and the variability in greenhouse gas (GHG) and fluvial fluxes is often governed by permafrost properties and local-scale hydrology and vegetation dynamics (11–13).

Despite their important role in global climate dynamics, peatland-specific processes remain unrepresented in broad-scale estimates of GHG feedbacks from thawing permafrost (10, 14–16). Permafrost thaw in ice-rich peatlands often occurs as abrupt thaw, resulting in thermokarst (ground collapse), a process that leads to wet postthaw environments with high rates of CH_4_ release (increasing radiative forcing). A dynamic global vegetation model (DGVM) that includes peatlands as well as permafrost, but not abrupt thaw and thermokarst processes, projects an increased C sink in permafrost peatlands under warming (7, 17). An inventory-based model suggests that abrupt thaw and thermokarst in high-latitude peatlands will trigger large losses of C to the atmosphere that are only partially offset by new vegetation and peat C storage (18).

Estimates of the total northern peatland C stocks remain variable and uncertain [300 to 600 Pg C (19); but see also ref. 20]. Our ability to assess peatland carbon–climate feedbacks is partly limited due to a lack of spatially explicit, observation-based maps of northern peatland extent and stocks of C and N. An inability to model present-day peatland C stocks has been a major source of systematic errors in Earth system models (ESMs) (21), which hinders predictions of changes in peatland C dynamics with future warming. A new generation of DGVMs is able to dynamically model peatland extent and C stocks but such models lack high-quality validation data and do not account for key permafrost processes such as thermokarst (4, 17, 22). Some previous map-based studies of soil C stocks have distinguished peatland coverage (23, 24), but these datasets lack explicit information on peat depth. Because these soil maps cannot separate peat layers from the underlying mineral subsoil, they are of limited use for understanding processes specific to peat formation and decay, and therefore future dynamics of peat.

Here, we aim to fill some of these knowledge gaps by mapping northern peatlands and assessing their vulnerability to permafrost thaw. We present maps of peatland depth and C and N stocks across the Northern Hemisphere (>23° latitude). These maps are based on peat core data compiled from multiple sources (*n* > 7,000 cores) that we combined with a range of global environmental datasets using a machine-learning approach. Estimates of peatland GHG and lateral flux budgets at steady state and under future permafrost degradation scenarios were derived from a spatial model based on meta-analyses of peatland flux observations combined with paleo-reconstructions. Using ensemble ESM projections of global warming stabilization scenarios, we provide spatially explicit projections of C and N dynamics and radiative forcing during peatland permafrost thaw and thermokarst development.

## Results and Discussion

### Mapped Peatland Extent and Peat C and N Stocks.

**Fig. 1.**
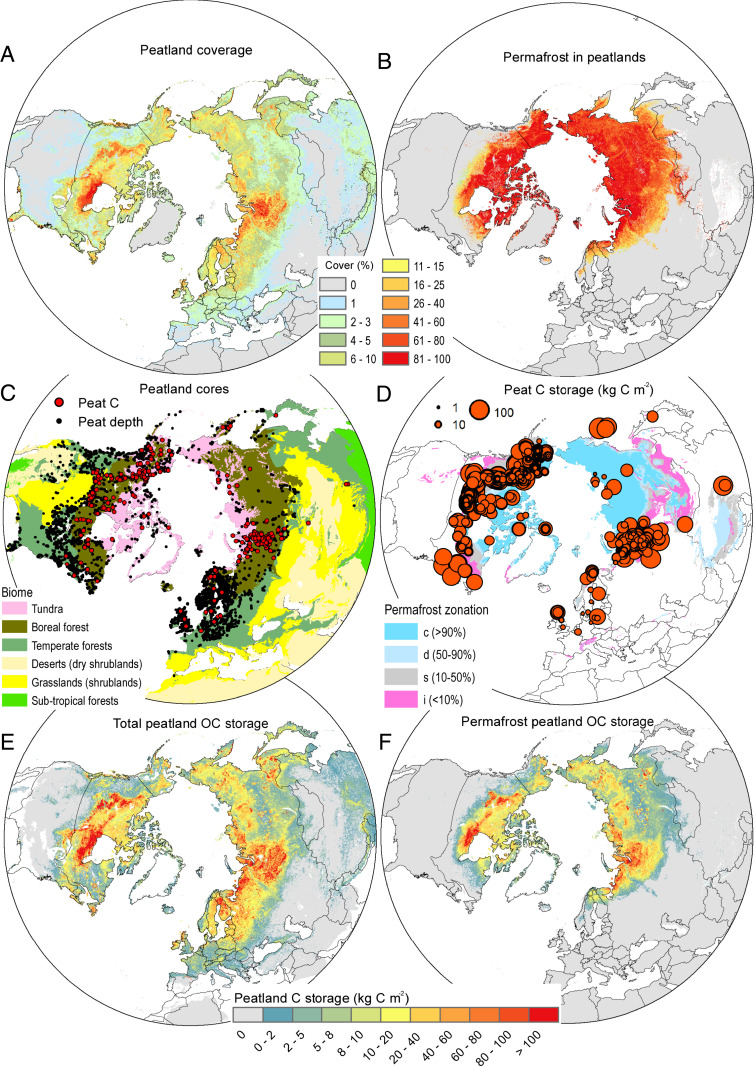
Peatland data and properties north of 23°N latitude. (*A*) Estimated areal coverage (in percentage) of peatlands based on the national soil inventory maps and SoilGrids250m. (*B*) Estimated areal coverage (in percentage) of permafrost in mapped peatlands based on the national soil inventory maps and SoilGrids250m, including a maximum threshold for permafrost at MAAT +1 °C (use the same legend as in *A*). (*C*) Spatial distribution of peat core sites with peat depth data (*n* = 7,111) and peat organic C storage (*n* = 782) over a map of biome distributions (biomes adapted from ref. 32). Sites with peat N stock data (*n* = 105) are not shown in the map (see Dataset S6), but are predominantly located in boreal forest and tundra biomes. (*D*) Sites with peat organic C storage data, with the size of site symbols proportional to measured peat organic C storage, over a map of permafrost zonation (33). (*E*) Estimated total peatland C storage and (*F*) permafrost peatland C storage.

Based on the peatland map products compiled in this study (25), we estimate northern peatland extent to be 3.7 ± 0.5 million km^2^ (mean ± root-mean-square error [RMSE]), including 1.7 ± 0.5 million km^2^ in permafrost (Fig. 1 *A* and *B*, Table 1, and *SI Appendix*, Table S1; peatlands defined as >40-cm surface organic soil material). This extent is similar to inventory-based estimates (1, 19, 26, 27) but suggests that both the global soil map WISE30sec (23) and the global PEATMAP dataset (28) underestimate northern peatland extent by ∼1 million km^2^. Our map is relatively consistent with the global maps in areas of very high peatland cover (e.g., West Siberian Lowlands and Hudson Bay Lowlands), but we identify more small peatland complexes outside of the core peatland regions (e.g., in central and eastern Siberia and the European Arctic west of the Ural Mountains). Our map of peatland extent was derived from the mean of two independent soil maps, which are the highest-resolution maps for delineating northern peatlands we know of, that is, harmonized national soil inventory maps (29, 30) and the global digital soil map SoilGrids250m (31). These maps separate peatlands with and without permafrost, but we include a maximum threshold for permafrost occurrence at mean annual air temperature (MAAT) of greater than or equal to +1 °C. See *SI Appendix*, supplement section S1 for detailed information on ground-truthing the peat extent maps using local observations.

**Table 1.** Summary of estimated peatland area and upscaled (area-weighted) peat depth, peat C storage, total peat C stock, peat N storage, and total peat N stock derived from the map products

	All peatlands	Permafrost-free peatlands	Permafrost-affected peatlands
Peatland area, million km^2^	3.7 ± 0.5	2.0 ± 0.5	1.7 ± 0.5
Peat depth, cm	249 ± 97	286 ± 111	205 ± 80
Peat OC storage, kg C⋅m^−2^	115 ± 41	123 ± 44	108 ± 39
Peat OC stock, Pg C	415 ± 147	230 ± 81	185 ± 66
Peat TN storage, kg N⋅m^−2^	3.1 ± 2.0	1.9 ± 1.2	4.2 ± 2.7
Peat TN stock, Pg N	10 ± 7.0	3.4 ± 2.3	7.1 ± 4.7

The reported uncertainty was assessed using point observations of peat depth, C and N stocks using a spatially weighted trimmed RMSE (5th/95th percentiles). For the peatland extent, the error is the propagated additive error of RMSE in relation to local ground-truthing maps (*SI Appendix*, Table S3) and variability between maps.

We determine peatland C stocks using a newly compiled dataset of peat cores with observations of peat depths (*n* = 7,111) of which some cores include peat organic C and N storage (*n* = 782 and 105, respectively) distributed across the Northern Hemisphere (Fig. 1*C* and Dataset S6). Based on the peat core data, the average peat C storage is 106 ± 66 kg C⋅m^−2^, ranging from 0.4 to 593 kg C⋅m^−2^ with substantial variability over short distances (Fig. 1*D*). From these, peat cores maps of peat C and N stocks (Fig. 1 *E* and *F*) are made by combining a machine-learning model of peat depth, the maps of peatland extent, and linear models for predicting peat C and N storage from peat depth (*SI Appendix*, Fig. S3). The machine-learning model of peat depth combines peat core data with spatial environmental data (summarized in *SI Appendix*, Table S2) and shows that underlying mineral soil texture, the extent of peatlands in the area, photosynthetically active radiation, and mean summer temperature were the most important variables for predicting peat depth (*SI Appendix*, Fig. S4). The maps show a mean peat depth across the circumpolar north (mean ± SD) is 249 ± 97 cm and that total peat C and N stocks are 415 ± 150 and 10 ± 7 Pg (mean ± RMSE), respectively (Table 1). We estimate that permafrost peatlands store 185 ± 70 Pg C and 7 ± 4 Pg N, a substantial part of the total stocks (Table 1). These estimates are largely consistent with most of previous estimates based on data aggregated from tables (1, 19, 26). However, the spatially explicit maps reveal patterns in peat C and peat N stock distribution that have been missed previously. We map larger extent of permafrost peatlands, but we also find these peatlands to be shallower with lower C stocks per area unit than previously assumed. Permafrost peatlands are on average ∼80 cm shallower than permafrost-free peatlands. This confirms that earlier local/regional findings of limited vertical peat accumulation in permafrost peatlands (34) are applicable across the permafrost region.

Combining our peatland C stock estimates with existing estimates for peatlands in the tropics [105 Pg C (35, 36) and the extratropical Southern Hemisphere (15 Pg C (2)], we estimate that peatlands store 530 ± 160 Pg C globally, with northern peatlands accounting for ∼80% of the total. The maps of northern peatlands reveal a very pronounced latitudinal pattern in peatland extent, with nearly half the global peatland C stored between latitudes of 60 and 70° N (*SI Appendix*, Fig. S7).

Our estimated peatland C stocks are difficult to reconcile with a recent estimate of >1,000 Pg C in northern peatlands (20). That study uses a conservative estimate of peatland areal extent (2.9 million km^2^) and argues that early onset of peatland expansion after deglaciation caused very high, sustained, C accumulation. Our approaches differ notably, e.g., in their inclusion of data from outside the northern peatland region, their lack of direct bulk density data, and their lack of any observational constraints to peat depth. To accumulate >1,000 Pg C in 2.9 million km^2^, mean peatland depths of ∼5 to 6 m are needed—twice as deep as suggested by our >7,000 data points. There is also a difficulty in reconciling a >1,000 Pg peatland C stock within the global carbon budget constraints offered by marine and ice-core paleo records of atmospheric CO_2_ concentration and isotopic composition (37), whereas our study can be reconciled with those top-down estimates. We also note that this high C stock estimate is currently being questioned elsewhere (37, 38).

### Present-Day Peatland C and N Balance.

Combining the peatland maps with syntheses of peatland annual flux and C accumulation observations, we calculate present-day peatland GHG fluxes as a sink of atmospheric CO_2_ at 0.10 ± 0.02 Pg C⋅y^−1^, a source of CH_4_ at 0.026 ± 0.002 Pg C⋅y^−1^, and a source of nitrous oxide (N_2_O) at 0.022 ± 0.005 Tg N⋅y^−1^. These are empirically based spatial estimates of northern peatland GHG balances, and the results are similar to previous estimates (3, 6). We estimate losses into aquatic systems (dissolved and particulate organic matter) to be 0.022 ± 0.02 Pg C⋅y^−1^ and 0.7 ± 0.5 Tg N⋅y^−1^. We note that the observational GHG data, for CO_2_ and especially N_2_O, remain very limited from northern peatlands.

Our estimated net C sink varies with MAAT. We find a significant relationship between MAAT and C accumulation rates over the past 2,000 y (logistic growth model, *n* = 129, *P* < 0.05, *R*^2^ = 0.3; *SI Appendix*, Fig. S8). By extrapolating this relationship spatially, we found an average accumulation rate of 34 g C⋅m^−2^⋅y^−1^ across the northern peatland region. We note that such long-term C accumulation estimates also implicitly include fire dynamics occurring naturally over time at these sites. Our meta-analyses reveal no clear climatic controls on CH_4_ and N_2_O fluxes; but these GHGs vary depending on peatland types (Dataset S1). The variability from different peatland types is accounted for by mapping distributions of permafrost peatlands, and estimating bog and fen cover from biome distributions (*SI Appendix*, Table S5).

As sinks of CO_2_, but net sources of CH_4_, peatlands cool the climate over long timescales. Using a snapshot of annual peatland flux, peatlands warm the climate over decadal time horizons, but cool it over longer time periods (39). Using a radiative forcing model (39), we estimate that the isolated radiative forcing from one year of present-day peatland GHG exchange peaks after 30 to 40 y at +0.075 W⋅m^−2^, mainly caused by net CH_4_ emissions (*SI Appendix*, Fig. S9). Over centuries, there is a net cooling, caused by CO_2_ sequestration through photosynthesis, which is reached after ∼350 y. These time constraints on radiative forcing are sensitive to the ratio of CH_4_ emission to CO_2_ uptake, which changes under peatland disturbances such as permafrost thaw.

### Peatland Permafrost Thaw and Postthaw C and N Balances.

**Fig. 2.**
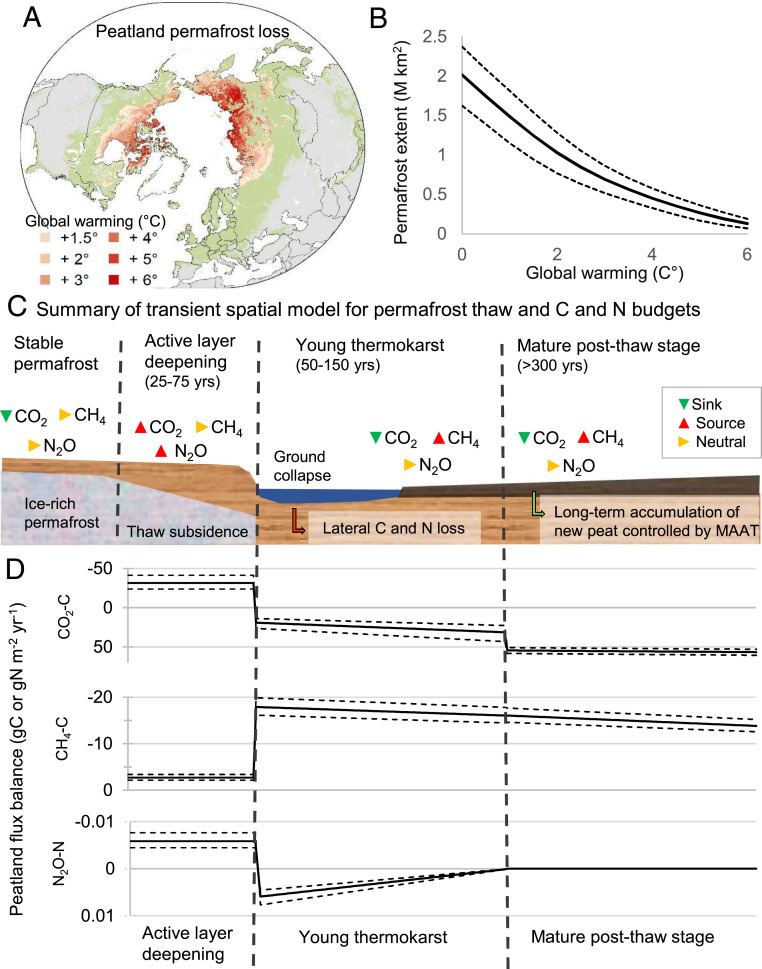
Projected permafrost loss from peatlands and the conceptual model of permafrost thaw impacts on GHG fluxes. (*A*) Map of projected permafrost loss from peatlands. Colors indicate temperature thresholds when equilibrium permafrost extent drops to less than 10%. (*B*) Projected equilibrium extent of permafrost in peatlands for global warming stabilization scenarios. Note that zero degree reflects preindustrial warming, with present-day climate already being close to +1 °C. (*C*) Spatial model of peatland transitions, including the properties and GHG balances of the different degradation and recovery stages. (*D*) Schematic mean (±SD) annual peatland GHG balances for the different stages of thaw and recovery (weighted average of all pixels). Negative numbers indicate C loss from peatlands, that is, a flux to the atmosphere (upward in the figure). Fluxes of CO_2_ for the transient permafrost thaw stages, as well as CH_4_ and N_2_O fluxes, are synthesized from observed fluxes in field or experimental studies (Datasets S1 and S5).

Based on an equilibrium model, we estimate that the preindustrial extent of permafrost in peatlands was ∼2 million km^2^, with a present-day coverage of 1.7 million km^2^. This area is projected to decrease to 1 million km^2^ at a 2 °C global warming stabilization above the preindustrial (Fig. 2*B*). At 6 °C global warming, we project that almost no peatland permafrost would remain. To model these permafrost losses, we used the present-day relationship between peatland permafrost extent and MAAT, extracted from maps (*SI Appendix*, Fig. S10), and projected it into future scenarios of global average warming stabilization above preindustrial (from +0.5° to +6 °C) using ensemble ESMs (Fig. 2*A*). This approach essentially adapts the method of ref. 40 specifically to permafrost peatlands. Because our approach was based on assumptions of equilibrium rather than transient responses processes, we cannot project how long it will take permafrost to thaw, but rather what the net, long-term effect will be.

To assess the effect of permafrost thaw on peatland C and N budgets, we distinguish four main potential stages in the long-term transition from stable permafrost to nonpermafrost peatlands (Fig. 2 *C* and *D*, Dataset S1, and *SI Appendix*, Fig. S11*A*). 1) Intact permafrost peatlands are sinks of CO_2_ and have near-neutral CH_4_ and N_2_O balances (41–44). 2) Gradual active layer warming and deepening cause releases of CO_2_ and N_2_O from the active layer and from newly thawed peat while CH_4_ remains near neutral (45–47). If thaw progresses into ice-rich permafrost, thermokarst may occur (18). 3) Young thermokarst stage fens and bogs are CO_2_ sinks and CH_4_ sources (44, 48, 49). There is no evidence of strong GHG losses from thawed peat (12, 50, 51), but chronosequence studies suggest large net losses of previously frozen peat (13, 18, 52), which we suggest may occur via dissolved or particulate organic C (DOC or POC) fluxes into aquatic ecosystems or through shorter transport and reposition. Young thermokarst lakes are sources of both CO_2_ and CH_4_ (53, 54). 4) Stabilization of postthaw peatland stages over centuries leads to weaker CH_4_ sources with time, and lakes that were CO_2_ sources transition into CO_2_ sinks (18, 54–56). We note that all four stages may not occur everywhere, and in many cases different stages may occur simultaneously across a peatland complex. In some sites, a permafrost peatland may experience, under drier conditions, a more extended stage 2 of thaw (active layer deepening) and then progress to a postthaw stage. The spatial model approximates this variability with probability distributions for different stages (*SI Appendix*, Fig. S11*A*). Our model framework allows us to explore postthaw C balances with simplified process representation, including the extent to which changes in peatland C stocks are attributable to active layer deepening or thermokarst expansion (18), but does not include other disturbances. Long-term data on C accumulation rates inherently include peat losses to fire, but our framework does not account for C and N losses from rapidly increasing peatland fire frequencies (8) or droughts (9). Our model does not account for landscape-scale hydrological impacts of thaw, such as increased hydrological connectivity (57) or increased evapotranspiration (58).

**Fig. 3.**
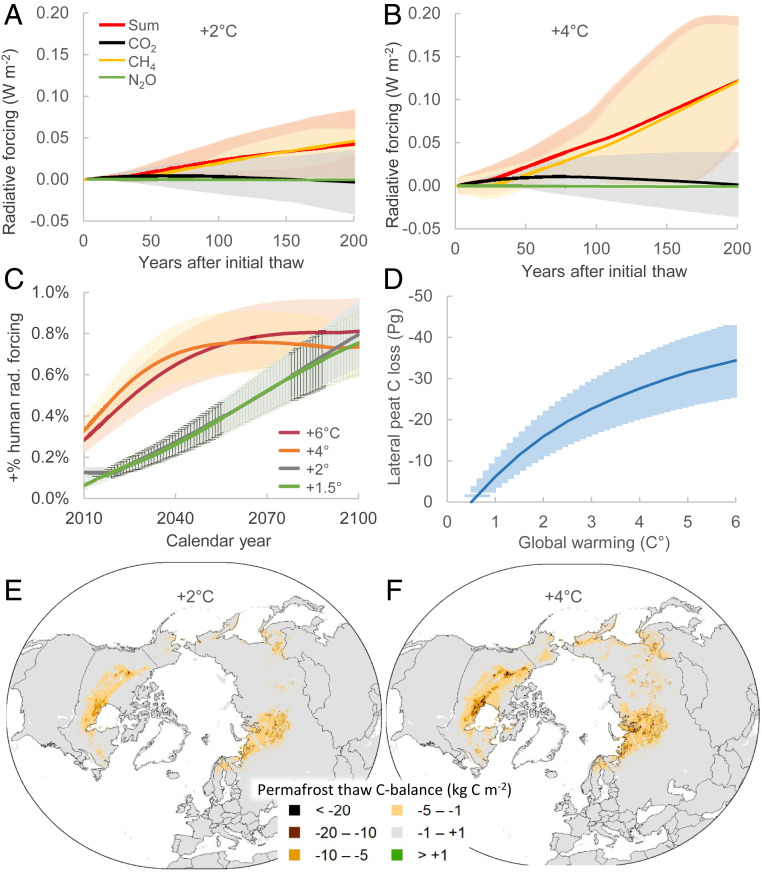
Projected GHG flux and radiative forcing from peatland permafrost thaw (calculated from the net change in GHG flux relative to stable peatlands) under different global warming stabilization scenarios. (*A* and *B*) Projected radiative forcing from GHG fluxes for three centuries for +2 and +4 °C global warming. (*C*) Added radiative forcing from peatlands in relation to human GHG emissions for this century, assuming that the +0.5 °C degree warming was passed in 1990 and starting the peatland thaw scenarios from that year (human radiative forcing consistent with the different warming trajectories) (60). (*D*) Projected gross lateral losses of peat C into aquatic systems (mainly as DOC/POC) inferred from permafrost thaw chronosequences. (*E* and *F*) Net peatland C-balance following the active layer deepening and young thermokarst thaw stages at +2 and +4 °C global warming.

Our modeling projects that permafrost thaw will cause a transient period of positive radiative forcing from peatlands, which will last one to three centuries (Fig. 3). This added radiative forcing is calculated from the difference between baseline peatland GHG balances at present climate and thaw scenarios. Under warming scenarios across the full range of +1.5 to +6 °C, transient losses of 2 to 6 Pg C as CO_2_ and CH_4_, but no significant losses of N_2_O are projected (Dataset S2 and *SI Appendix*, Fig. S11*B*). Two centuries after initial thaw, the combined radiative forcing of this transitional GHG release reaches 0.05 and 0.13 W⋅m^−2^, respectively, for +2 and +4 °C global warming scenarios (Fig. 3 *A* and *B*). The radiative effect is mainly caused by CH_4_ release. For CO_2_, initial release from peat decomposition during active-layer deepening is compensated by a net sink effect in the thermokarst stage. We project smaller CO_2_ losses, but similar fluxes of methane CH_4_, compared to a previous study of abrupt thaw (18). We estimate a minimal radiative forcing contribution from N_2_O, allaying concerns of potential added N_2_O forcing from peatland thaw (46). After long-term stabilization of the thaw-pulse (>200 y), the peatlands will be an annual net sink of C, a source of CH_4_, and near neutral for N_2_O (*SI Appendix*, Fig. S11*C*). Both the strength of the CO_2_ sink and the CH_4_ source increase with MAAT. This is because a warmer climate increases the peatland CO_2_ sink capacity (due to higher plant productivity), but also increases the fraction of peatlands that change from dry permafrost peatlands to wetter permafrost-free peatlands, thus increasing CH_4_ emissions. Warmer and wetter climates in the future may also lead to the formation of new peatlands, and C accumulation, in high-latitude regions (4, 59), but this is not addressed in our model calculations.

To assess the societal relevance of the projected GHG fluxes, we compare estimated net added GHG fluxes from the permafrost thaw to full anthropogenic radiative forcing scenarios consistent with United Nations Framework Convention on Climate Change Conference of the Parties negotiations (60). Across the full range of +1.5 to 6 °C global warming, northern peatland emissions amount to adding +0.4% to +0.8% onto the forcing from projected anthropogenic emissions by the year 2050 (Fig. 3*C*). For the low-emission scenarios, the contributions from permafrost thaw in northern peatlands peak near +2% of human emissions, but not until the next century.

### Losses of Deep Permafrost Carbon and Its Potential Fate in the Aquatic System.

In addition to GHG losses to the atmosphere, we project additional net cumulative lateral losses to aquatic systems of 10 to 30 Pg C and 0.4 to 1.1 Pg N from permafrost thaw under +1.5 to +6 °C global warming (Fig. 3 *D*–*F*). These projections are based on spatial modeling of synthesized peatland permafrost-thaw chronosequences, which have shown rapid (decadal) postthaw losses of deep permafrost peat (13, 18, 61) (*SI Appendix*, Table S7). These estimates are based on only five chronosequence studies and are more speculative than our other projections. Similar data have been used to infer C losses as CO_2_ (18), but because GHG flux observations do not seem to support thaw-induced losses of this magnitude (Dataset S1 and refs. 12, 50, 51), we suggest these would most likely occur laterally. This putative lateral loss may occur as DOC or POC fluxes into aquatic ecosystems or through shorter transport and reposition in adjacent ecosystems. If correct, such losses of peat C could have significant implications for aquatic biogeochemistry and ecosystems (62). Averaged over space and time, our modeled losses add up to 11 to 18 kg C⋅m^−2^ per unit of thawed peatland over the full ∼100-y postthaw period. Although limited, there are observations of lateral flux from thawing permafrost peatlands against which these numbers can be compared (*SI Appendix*, section S4), and we conclude that fluvial losses of these magnitude can be supported by data from West Siberia (63, 64) but not from a Boreal peatland dominated catchment in western Canada (65). Our projected total net gaseous and lateral C losses are 5 to 10 Pg C higher than those estimated in ref. 18, a difference mainly attributable to a higher projected area of permafrost thaw and the inclusion of active layer deepening losses in this present study. We note that large fractions (half or more) of C transported as POC/DOC may be degassed as CO_2_ directly from inland water surfaces (63, 66), potentially increasing the total atmospheric burden caused by permafrost thaw in peatlands.

## Conclusions

Our maps of northern peatlands are valuable tools for quantifying the role of peatlands in the global C and N cycles as well as for assessing peatland vulnerability to permafrost thaw. Further insights into the fate of northern peatlands under a changing climate can be gained from applying them in spatial studies of peatland fire or drought dynamics. Northern peatlands hold ∼80% of the global peatland C and N stocks and are presently a sink of atmospheric CO_2_ (0.1 ± 0.02 Pg C⋅y^−1^). However, the potential for peatlands to remain long-term C sinks may reverse due to permafrost thaw. If the global climate stabilizes at 2 °C warming, we estimate that only a half of the preindustrial permafrost peatland extent will remain; but at +6 °C warming, peatland permafrost essentially disappears. This thaw mobilizes the large currently frozen C and N stocks for decomposition. Methane dominates the radiative forcing from peatland permafrost loss, with a complex transient response of CO_2_ and minor contributions of N_2_O. The projected radiative forcing from direct peatland GHG emissions remains below 2% of human CO_2_ emissions.

Projected cumulative permafrost peatland C loss shows particularly vulnerable regions close to the southern margins of permafrost distribution. While observations of permafrost thaw effects remain scarce, our projections are consistent with observational evidence. Presently, broad-scale permafrost thaw is evident at +1 °C above preindustrial temperatures (67), and the effects on permafrost peatland extent and GHG release have already been observed for several decades (56, 68, 69). Widespread increase in thermokarst has been observed even in very cold permafrost (70). Observations from streams and rivers draining thawing peatland areas show mixed responses, with limited lateral losses in some sites but other data supporting scenarios of large lateral C losses into fluvial systems (63, 64). Our projections of permafrost peatland thaw causing net C losses over several centuries are contrary to some modeling studies (7, 17), but at present DGVMs or ESMs cannot simulate abrupt permafrost thaw. Similar to earlier studies, our data-constrained spatial modeling approach is limited by the scarcity and variability of permafrost peatland C and N flux data (18). The specific processes of peatland permafrost thaw we study here have been unaccounted for by previous spatial estimates of the permafrost carbon feedback. Our projected combined gaseous and aquatic C losses would add 30 to 50% onto previous spatially explicit estimates of permafrost-C losses under warming where abrupt thaw and peatland thaw was not quantified (10, 14).

## Methods

The mapping and analyses of northern peatland properties and future vulnerability was based on compilation and analyses of peat core data, compilation and analyses of peatland extent from soil maps, upscaling peat depth, C stocks, and N stocks to the full region, compiling data on observed C and N balances under permafrost thaw, compiling paleo-evidence for long-term C balances, and scenarios projecting/modeling C and N balances at present and under permafrost thaw for different global warming scenarios. Here, we present a summary of methods and data sources, but see *SI Appendix* for a more detailed description of all methods and data sources.

### Peat Core Data and Maps of Peatland Spatial Extent.

A total of 7,111 geolocated peat cores with peat depth data was compiled (Dataset S6). Only sites where basal peat was reached are included. A subset of 782 cores have data on peat organic carbon content (OC% by weight) and dry bulk density. A subset of 105 cores has additional data on peat total N content (weight % N). The sources of data were from refs. 26, 71–75, and previously unpublished data.

This study based estimates of peatland spatial extent on soil classification maps. The study region is limited to the extratropical northern hemisphere (defined as north of 23° latitude). Three different map products were used or evaluated for their capacity to accurately map peatland extent: the global WISE30sec dataset (23), the global SoilGrids250m dataset (31), and harmonized national and regional soil maps (29, 30). We refer to these references for details about how the maps were made. The WISE30sec dataset was not used for peatland mapping as it had too low resolution. The SoilGrids250m and the national/regional soil maps were combined and harmonized for this study; see *SI Appendix* for more details. All datasets were projected using equal area projections and were resampled to 5-km grids using bilinear interpolation.

### Spatial Analyses and Upscaling of Peatland Properties.

The spatial scaling of peat depths was carried out using random forest machine learning (RFML). Random forest is a tree-based machine-learning method that uses bootstrapped samples (here peat cores) to grow a large number of decision trees (*n*_tree_) with randomized environmental predictors at each tree node (*m*_try_). These trees are then averaged to predict new data (76, 77). A RFML model, with 1,000 trees (*n*_tree_), was trained using the observational data. In total, 6,038 peat cores had sufficiently precise geolocation and matched point-to-pixel overlays for all of the environmental training variables (*n* = 12; *SI Appendix*, Table S2). We used a 10-fold cross-validation with five repetitions providing *m*_try_ as a tunable parameter for model training using the *caret* package in R (77, 78). We applied bias correction to the predicted peat depths using best angle residual rotation of the peat depth map (*SI Appendix*, Fig. S6, and ref. 79).

By combining the RFML model of potential peat depth with the map of peat coverage, we calculated area-weighted peat depths and peat volumes. To calculate stocks, the modeled peat depths were used to estimate peat organic C and N storage (kilograms of C or N per square meter) using linear relationships formulated based on the peat core data (*SI Appendix*, Fig. S3). The estimated C and N storage was then used to calculate total C and N mass per pixel. Uncertainties are reported as RMSE based on 5th/95th percentiles of residuals between modeled and observed values of peat depth.

### Scaling C and N Balances and Projecting Permafrost Thaw.

The baseline C and N balances of peatlands, including GHGs (CO_2_, CH_4_, and N_2_O), were estimated based on paleo-reconstructions of C balances as well as syntheses of flux measurements from permafrost- and permafrost-free peatlands (Dataset S1). Paleo-observations were used for long-term net C budgets and syntheses of GHG flux measurements for shorter time intervals in projections of thaw. We developed a simple spatially explicit inventory model to assess the impact of peatland permafrost thaw scenarios on the stocks of C and N as well as GHG fluxes.

The applied permafrost thaw scenarios (*SI Appendix*, Fig. S2*A*) assume that, once the temperature threshold for thaw is crossed, the peatlands are affected by active-layer deepening for a period of ∼25 to 75 y (with a mean of 50 y) until the thaw progresses into ice-rich, deeper peat. This time period was calculated based on active-layer deepening of 1 cm per year (estimated from refs. 46, 80) and that the average depth to ice-rich peat from the bottom of the active layer in permafrost peatlands is ∼25 to 75 cm (calculated from data in refs. 72, 81–83). If thaw progresses into the ice-rich core of the permafrost peatland, thermokarst (ground collapse) occurs. Postthaw thermokarst peatlands or lakes were assumed to gradually transition to mature thermokarst systems over 50 to 150 y (a mean of 100 y). This time period of transition into mature thermokarst was based on an average of studies on postthaw chronosequences, which suggested somewhat longer transition times of ∼150 to 200 y (estimated from refs. 13, 18, 54, 61) and remote-sensing studies that showed substantial lake drainage or fen-vegetation infilling in some areas over periods of a few decades (56, 83).

For flux scaling, we separated nonpermafrost and permafrost peatlands from postthaw peatlands. All classes were further separated into minerotrophic and ombrotrophic peatlands, but only if there were statistically significant differences in C accumulation rates or GHG balances. The spatial extent of minerotrophic and ombrotrophic peatlands was scaled from the Canadian Peatland Map (84), as fractions within tundra, boreal, and other biomes (includes temperate, oceanic, mountain, and prairie climate regions; biome distributions from ref. 32; *SI Appendix*, Table S5).

### Calculations of C and N Balances.

The C balance of stable peatlands was modeled based on observed long-term apparent C accumulation in the late Holocene (last 2,000 y) from northern (*n* = 122; ref. 26) and tropical (*n* = 7; ref. 2) peatlands (Dataset S4). The best model fit was achieved with a logistic model (S-shaped curve) that is able to model growth with saturation at both high and low temperatures (85) (*R*^2^ = 0.3, Akaike information criterion = 28,822; *SI Appendix*, Fig. S9*B*). This model predicted mean potential C accumulation scaled for the extratropical northern hemisphere under present baseline climate is 34 g C⋅m^−2^⋅y^−1^.

The CO_2_-C fluxes during thaw stages were based on a meta-analysis of full year budgets from thawing permafrost in the literature (*SI Appendix*, section S1.5 and Dataset S1). The net C budget following permafrost thaw was based on chronosequence studies of postthaw permafrost peatlands (13). Old permafrost C is lost following thaw, while increased ecosystem productivity in the young thermokarst (postcollapse) means that the surface peat is gaining C. In the early thaw stages, the loss of old C is much more rapid than the gain of new C. The loss of old permafrost C can be estimated as a function of prethaw C stock (13, 18) (*SI Appendix*, Table S7). The C loss during the first 100 y after thaw was estimated from the peatland C stock maps using the simplified equation *y* = 1.1451*x*^−0.0771^, where *y* is the fraction of prethaw C that is lost in 100 y after thaw and *x* is the stock of prethaw C in kilograms of C per square meter (*R*^2^ = 0.93, from 100 y in *SI Appendix*, Table S12). We scaled the changes in N pools from the C pools based on typical C:N ratios of permafrost peatlands and nonpermafrost peatlands in tundra regions and boreal regions (*SI Appendix*, Table S6).

All data for estimated CH_4_ fluxes were from a recent synthesis of year-round CH_4_ fluxes in northern wetlands (86). We used only sites with organic soils and separated nonpermafrost, permafrost, and postthaw sites. We further distinguished the minerotrophic peatlands (swamp, marsh, and fen classes, following the Canadian wetland classification system) from ombrotrophic peatlands (bogs).

For minerotrophic and ombrotrophic permafrost-free peatlands, we used annual N_2_O budgets from a synthesis of N_2_O fluxes from northern soils (87). Annual/seasonal N_2_O data from Arctic peatlands are limited to a single site located in western Russia with discontinuous permafrost. We used published N_2_O flux data from this site (41, 88) as N_2_O emission estimates for minerotrophic and ombrotrophic (bare and vegetated) permafrost peatlands (Dataset S1). Data on N_2_O (46) and CO_2_ (47) fluxes from peat mesocosms during simulated permafrost thaw were used to develop a scaling ratio of N_2_O release relative to C release (see *SI Appendix* for more details).

### Model of Permafrost Fraction in Peatlands.

The model of permafrost fraction in peatlands was derived using the method developed in ref. 40, where a relationship between permafrost fractional coverage and MAAT was fitted by minimizing RMSE between MAAT and mapped permafrost fraction in peatlands. The equation used is as follows:

$$\text{Permafrost}\;\text{fraction}=0.5f_{\max}\text{ERFC}\left(\frac{\text{MAAT}+\mu }{\sqrt{2\sigma^{2}}}\right),$$

where ERFC is the complementary error function (using the *pracma* R package).

As in ref. 40, the curve was refitted using “maximum” and “minimum” permafrost fraction to give upper and lower estimates of permafrost fraction, as well as a central estimate. The maximum and minimum extents were derived from the highest and lowest per-pixel estimates of permafrost fraction in the national polygon maps and SoilGrids, respectively. Thus, three different parameter values (for central, upper, and lower curves, respectively) were fitted for μ (1.95, 0.7, and 3.1), σ (7.35, 6.1, and 4.5), and *f*_max_ (0.92, 0.96, and 0.86). We assumed that mapped permafrost extent at +0.5 °C global warming (relative to preindustrial levels) was in quasi-equilibrium with the climate of the 1960 to 1990 period, which we also consider to be representative for the permafrost extent in the maps. Global warming stabilization scenarios at 0.5 °C intervals up to a maximum of 6 °C were used for the future projections.

### Modeling Radiative Forcing.

The projected GHG budgets, including CO_2_, CH_4_, and N_2_O fluxes, from the spatial model were used to calculate the future radiative forcing effect. A range of GHG flux scenarios (available in Dataset S3) were exported from the spatial model and used as input in a radiative forcing model (39), with additional parameterization for N_2_O and modifications to atmospheric CO_2_ lifetimes (89). Separate GHG flux scenarios were calculated for stabilized permafrost conditions at 0.5° increments from 0° to +6 °C global warming stabilization (background concentrations were stable anthropogenic present-day emissions). Separate runs were also done for fluxes resulting from the transient thaw scenarios for each incremental warming, with the added radiative forcing from permafrost thaw calculated from the net changes in GHG fluxes relative to stable baseline peatland GHG balances at present (Fig. 2 and Dataset S1). The net radiative effect of the transient thaw is calculated as the difference between stable scenarios and the transient scenarios. To compare the magnitude of permafrost–peatland thaw emissions to anthropogenic emissions, radiative forcing from anthropogenic emissions together with peatland thaw emissions were compared to anthropogenic emissions alone. The projections for anthropogenic emissions were retrieved from Climate Scoreboard (60) and are computed using the C-ROADS climate policy model (90). For these calculations, we assume that +0.5 °C global warming is consistent with peatland fluxes in 1990 to 2000 (assuming decadal lags in thaw from the 1960 to 1990 climate normal) and that +1 °C warming is consistent with present day.

## Supplementary Material

Supplementary File

Supplementary File

Supplementary File

Supplementary File

Supplementary File

Supplementary File

Supplementary File

## Data Availability

The results and peat core data are summarized in Datasets S1–S6. Maps of predicted peatland extent, peat depth, and peat C and N storage (10-km pixels) are archived and freely available for download at https://bolin.su.se/data/hugelius-2020.
