# Supplementary material for: Large stocks of peatland carbon and nitrogen are vulnerable to permafrost thaw
Source: Proc Natl Acad Sci U S A. 2020 Aug 10;117(34):20438–46. doi: 10.1073/pnas.1916387117 (PMC7456150; doi:10.1073/pnas.1916387117)
Supplement: Supplementary File [file pnas.1916387117.sapp.pdf]

Supplementary Information for

Large stocks of peatland carbon and nitrogen are vulnerable to permafrost thaw

Gustaf Hugelius, Julie Loisel, Sarah Chadburn, Robert B. Jackson, Miriam Jones, Glen MacDonald, Maija Marushchak, David Olefeldt, Maara Packalen, Matthias B. Siewert, Claire Treat, Merritt Turetsky, Carolina Voigt and Zicheng Yu

Corresponding author: Gustaf Hugelius, Department of Physical Geography, Stockholm University, 106 91 Stockholm, Sweden.
Email: gustaf.hugelius@natgeo.su.se.

This PDF file includes:

- Supplementary text
- Figures S1 to S11
- Tables S1 to S7
- SI References

Other supplementary materials for this manuscript include the following:

- Datasets S1 to S6, found in a separate MS Excel documents
- Maps and spatial databases are archived and freely available at https://bolin.su.se/data/

www.pnas.org/cgi/doi/10.1073/pnas.1916387117

Supplemental text

S1. Extended method

This text provides a longer and more detailed method description, there is substantial overlap with the main method section to allow for easier reading of this text. The mapping and analyses of northern peatland properties and future vulnerability was based on compilation and analyses of peat core data (Section 1.2 below), compilation and analyses of peatland extent from soil maps (section 1.3), upscaling peat depth, C stocks and N stocks to the full region (section 1.4), and syntheses of flux and paleo-data supporting scenarios projecting C and N balances at present and under permafrost thaw for different global warming scenarios (section 1.5).

S1.1 Summary of spatial and statistical analyses

All spatial analyses were performed in the software ArcGIS 10 (ESRI, Redlands, CA, USA). Statistical analyses were performed in PAST 3.1 (1), R (2) and ArcGIS 10 (ESRI, Redlands.CA, USA). All spatial analyses were performed in equal-area or equidistant geographic projections suitable for the scale and extent of the analyses. Mean values of statistical samples are reported with ±1 SD. Upscaled estimates that combine measurements with spatial or linear modelling are reported with error estimates that are either 95% confidence intervals or, if assessed from validation data, root mean square errors (RMSE). Statistical tests were two sided, considered significant if $p<0.05$, applying a false discovery rate correction when appropriate.

S1.2 Peat core data

In total, 7111 geolocated peat cores with data on full peat depth were compiled. A subset of 782 cores have data on peat organic carbon content (OC% by weight) and dry bulk density. A subset of 105 cores have additional data on peat total N content (weight % N). The sources of data were refs. (3–8) and previously unpublished data. The supplementary material contains all point data and includes a reference to the source of each data point. This data compilation includes all major data sources known to the authors, and is thus the most comprehensive dataset available at this time. The spatial distribution of data is clustered and the data represents various sources collected using varying methods for different purposes over several decades. These properties of the data make their use for spatial scaling more challenging than if the data had been systematically collected for purposes of spatial scaling (using e.g. randomized or stratified sampling designs).

Because these data points were not collected for peat depth modelling, there may be sampling biases caused by scientists wishing to core the center of peatlands for other purposes (e.g. finding the oldest peat for paleo-reconstructions). The problem of peat depth sampling biases has been extensively discussed in references (9–11). In smaller isolated peatland basins, the spatial variability in peat depth can be substantial (11). However, for two reasons we believe that the influence of this bias on our results is relatively limited. First, larger peatland complexes in the main northern peatland regions (such as the West Siberian Lowlands and Hudson Bay Lowlands) are often overlying relatively flat topography. Second, much of the input data is from national surveys that actually include transects across individual peatland complexes and thus capture this variability. With such survey-data included, the machine learning approach can partly offset the sampling bias, by combining environmental variables from different locations and different depths. Thus for a peatland with only one point, information from other sites that have more data (and shallow peat) will still affect the outcome. This can partly explain the trend of more frequent deep residual differences between predicted and observed peat depth south of the core peatland regions (fig S6; these sites tends to be isolated basins with less data available).

S1.3 Maps of peatland spatial extent

This study based estimates of peatland spatial extent on soil classification maps. The study region is limited to the extratropical northern hemisphere (defined as north of 23° latitude). Three different map products were used or evaluated for their capacity to accurately map peatland extent: the global, broad scale, WISE30sec dataset (12), the global SoilGrids 250 m dataset (13) and harmonized national and regional soil maps (4, 14). Other datasets, such as the GLWD (15) or PEATMAP (16) datasets also include spatially explicit estimates of peatland cover. These datasets were downloaded and carefully examined. However, becuase these datasets are not internally consistent or harmonized (i.e. they use a patchwork of different sources) they therefore not used further or considered for peatland mapping in this study.

The two global scale products (SoilGrids and WISE30sec) were downloaded, projected to equal area projections and resampled to 5 km grids. The SoilGrids 250 m dataset is based on extensive machine learning algorithms and includes soil classifications following the USDA Soil Taxonomy which was

used for this purpose. The peatland extent (organic soil extent) in WISE30sec dataset is from the HWSD and can be traced back to the original FAO soil map of the World (17).
The regional soil maps were combined and harmonized for this study. Such maps are available for the geographic regions Canada, Europe (except Italy), Mongolia, USA and Russia. The soil maps used were the Northern and Mid-latitude soils maps (14) and the NCSCDv2 (10, 18). We refer to these references for details about how the maps were made. These two databases were projected to equal area projections and merged, preserving NCSCDv2 where they overlapped. The dataset was checked for consistency and adjusted so that the various mapped coverages added up to 100% (case by case solution applied, whenever the source of error was not evident water cover was adjusted, but preserving the relative coverage between other classes). Sliver polygons where removed by identifying those polygons that had an area <5000 m^2 and a circularity index value of <0.01. The extent of mapped peatlands and permafrost in peatlands in the maps was evaluated against individual peat cores and local-scale high resolution maps (see supplement).

S1.4 Spatial analyses and upscaling of peatland properties

S1.4.1 Spatial upscaling of peat depth

Upscaling of point observations to areal coverage can be made either based on thematic mean upscaling (e.g. mean values for different regions, soil units or landscape units) or some form of digital mapping method. The peat depth dataset is highly spatially clustered and exhibits characteristics that makes it unsuitable for analyses with predictive interpolation methods that rely on semivariogram modelling, such as kriging or co-kriging. This conclusion was based on extensive examination of the properties of the data, including semivariograms at multiple distances, which consistently showed a lack of local-scale autocorrelation. While Geographically Weighted Regression (GWR) can work well on spatially clustered data it can be sensitive to limited local-scale spatial autocorrelation in the data. Machine learning methods have been shown to produce relatively robust digital maps with a wide range of input data, even with spatially clustered or noisy data (19).
Random Forest Machine Learning (RFML) has been identified as a suitable machine learning method for the spatial interpolation of environmental variables and several studies have shown its successful and often superior application in digital soil mapping (20–22), including peatland depth and SOC stocks (23, 24) . RFML is a tree based machine learning method that can use an ensemble of bootstrapped trees for regression estimates (25). In RFML each regression tree depends on a random selection of environmental predictors at nodes. The ensemble of all trees are then averaged for prediction. We selected 1000 trees (n_{tree}). A great advantage of tree based machine learning methods is their ability to cope with non-linear relationships between the predicted variable and covariates representing the environmental space (26). This makes them particularly suitable for regional to global mapping approaches. Modelling was performed using R statistical software (2), following standard procedures as outlined in ref (27). The RFML was applied to the 12 environmental predictor variables (Table S2) and a subset of the peat depth cores for which it was possible to extract corresponding values of the environmental variables. The model was then trained using 10-fold cross-validation with five repetitions to develop a stable model using the *caret* package (28). The best performing model was automatically selected based on the lowest RMSE (142.2 cm) corresponding to an R^2 0.304 at a m_{try} value of 3. Predictor variables reflecting soil parent material texture, mean summer temperature and pixel peatland cover have the highest variable importance in predicting peat depth at circumpolar scale (Fig. S4). To reduce an observed regression to mean effect in the original RFML model, we applied bias correction using residual rotation on the model, by adapting an existing approach to raster maps (29). The effect on observed against predicted peat depth in values of the raster map of peat depth is presented in figure S5. The residual rotation decrease the MSE from 9984 to 9252 and improved Lin's concordance correlation coefficient from 0.767 to 0.868, which is a measure of accuracy and precision of agreement to a 45° line (30).
Because of the very high variability in peat depth, and problems with some imprecise geolocations of point data, the spatial resolution of the analyses was reduced to 10 km pixels (from an original 5 km resolution). Data users should note that the maps are generalized and meant to show mean peat depths regionally. As with all generalized and broads-scale maps, it is not appropriate, or good scientific practice, to relate information from a single site or peat core to the generalized 10*10 km pixel values. There is sometimes large differences between individual peatland cores and the mean mapped depths, which is evident from a map of residuals between observed and predicted peat depths (Fig. S6).

S1.4.2 Calculating peat depth, volume and stocks of C and N

By combining the RFML model of potential peat depth with the map of peat coverage we calculated area-weighted peat depths and peat volumes. To calculate stocks, the modelled peat depths were used to estimate peat organic C and N stocks (kg C or N per m^2) using linear relationships formulated based on the peat core data (Fig. S3). The linear functions to estimate peat C and N stocks from peat depth were done separately for permafrost-free and permafrost peatlands, respectively. The linear models were fit on log-transformed data using Major Axis (MA) linear models (31) (considered robust with limited data) with intercepts set to zero (Fig. S3, see supplement section S2 for more details). The estimated C and N stocks were then used to calculate total C and N mass per pixel.

S1.4.3 Uncertainties in estimates of peat depth, volume and stocks of C and N

In addition to the 10-fold cross validation of the RFML map we also performed a consistent uncertainty estimate of peat depth, volume and stocks of C and N against the full point datasets for depth, C stock and N stock. For each variable, we calculated the root mean square error (RMSE) between observed and modelled values for all observations. We also calculated trimmed RMSE (based on 5th/95th percentiles of residuals), R^2 between modelled and observed values and deviation of the mean in modelled and observed. Given the very high variability in the point data (also over short distances), the trimmed RMSE was considered as the most robust estimate of error and this is reported in the main text and used to calculate uncertainty ranges for upscaled peat volume, C mass and N mass in Pg.

S1.5 Scaling C and N balances and projecting permafrost thaw

The baseline C and N balances, including GHGs (CO_2, CH_4 and N_2O) of peatlands were estimated based on paleo-reconstructions of C balances as well as syntheses of flux measurements from permafrost- and permafrost-free peatlands (Dataset S1). Paleo-observations were used to estimate constrain long-term net C budgets. Paleo-observations have the advantage of integrating long time periods and, thus, can be used to constrain long-term C budgets in a way that flux observation time-series cannot. Another advantage of the paleo-observations is that they account for the total C and N losses including both atmospheric and aquatic losses, while most of the flux studies report just one of the two components. Syntheses of flux measurement data were used to estimate GHG balances and lateral losses (32) for shorter time intervals (such as post-thaw thermokarst) and to help attribute long-term changes in bulk C stocks to gaseous or lateral (hydrological) fluxes.

We used a spatial model to assess the impact of peatland permafrost thaw scenarios on the stocks of C and N as well as GHG fluxes. The values, approaches, and data sources are summarized in Figure 2 and Dataset S1 of the supplement, but also described below. Permafrost peatland hydrology is locally complex and variable, with thaw causing both drying and wetting depending on local peatland morphology (33, 34). We note that such complexities cannot yet be mechanistically accounted for; it would require couple process models with access to extremely high-resolution maps of peatland, soil and ground-ice properties. Here, we use a simplified conceptual model to represent the main stages of thaw and represent spatial and temporal uncertainty by statistical uncertainty range assessment and probability distributions. The applied permafrost thaw scenarios (Fig 2a) assume that once the temperature threshold for thaw is crossed, the peatlands are affected by active layer deepening for a period of ca. 25-75 years (mean 50 years) until the thaw progresses into ice-rich, deeper peat. This time period was calculated based on active layer deepening of 1 cm per year (estimated from refs. (35, 36) and that the average depth to ice rich peat from the bottom of the active layer in permafrost peatlands is ca. 25 to 75 cm (calculated from refs. (9, 37, 38).

If thaw progresses into the ice-rich core of the permafrost peatland, thermokarst (ground collapse) occurs. Post-thaw thermokarst peatlands or lakes were assumed to gradually transition to mature thermokarst systems over 50-150 years (mean 100 years). This time period of transition into mature thermokarst was based on an average of studies on post-thaw chronosequences which suggested somewhat longer transition times of ca 150-200 yrs (estimated from refs. (39–42)) and remote-sensing studies that showed relatively rapid lake drainage or fen-vegetation infilling in some areas ((43, 44). Due to the large imprecision of these time-transition estimates, we generated wide probability distributions for these time phases which span a wide range of possible occurrences, this means that the active layer deepening phase could, in principle, last anywhere from 1 to 150 years and the young thermokarst phase from could occur already at year 1 after thaw and proceed up to 250 years (Fig S11). The uncertainty from the time ranges were propagated into the estimate of upscaled flux uncertainties. Also, during these stages there is variability in soil drainage conditions (wetness) which, in turn affects the GHG-balance (45). We cannot explicitly account for this fine-resolution variability, but we propagate the added uncertainty from this into the estimate of upscaled flux uncertainties. This is based on ref. (46) who in their thaw experiment included active layer deepening under both dry and

wet conditions, from their data, we find that the range between wet and dry condition adds +24% to the flux uncertainty range.

The last phase, mature thermokarst, transitioned over several centuries to stable peatlands (assuming with an equal coverage of minerotrophic and ombrotrophic peatlands in mature peatlands). The time constraint on this transitional period is very poorly known and in this study it was assumed to exceed 300 years. For the calculations of transitional flux rates it was set to 750 years (500-1000 year range following ref. (41)), but this assumption has a limited effect the results in the main text or figures since the projections are not reported beyond 300 years.

In the scaling of fluxes, we separated non-permafrost and permafrost peatlands from post-thaw peatlands. All classes were further separated into minerotrophic and ombrotrophic peatlands, but only if there were statistically significant differences in the respective C accumulation rates or GHG balances. The mapping of minerotrophic and ombrotrophic peatlands is challenging as there are no global or hemispheric maps of these properties. But there is a regional-scale map over Canada (47) and we assumed that the general patterns of peatland morphology across biomes in Canada were broadly representative for the whole study area. The spatial extent of minerotrophic and ombrotrophic peatlands was scaled from the Canadian Peatland Map (47), as fractions within Tundra, Boreal and other biomes (includes Temperate, Oceanic, Mountain and Prairie regions; biome distributions from ref. (48). This data is summarized in table S5.

S1.5.1 Calculations of C and N balances

The CO_2 fluxes and lateral fluxes of *C* and N in peatlands were assessed using a combination of approaches. In stable peatlands (with or without permafrost), the baseline apparent long-term C accumulation was modelled based on MAAT, while lateral C losses as dissolved organic carbon (DOC) in aquatic systems were estimated based on literature (Dataset S1). The CO_2-C flux was calculated as the residual between long-term C accumulation, lateral C flux and CH_4-C flux. In the permafrost thaw scenarios, CO_2-C fluxes are based on a literature meta-analyses of measured annual fluxes from thawing permafrost (see table S4) while the net loss of old peatland C was modelled from observations from paleo-reconstructions in post-thaw chronosequences (see section 1.5.1.2).

S1.5.1.1 Estimating long-term apparent C accumulation rates

The C balance of stable peatlands was modelled based on observed long-term apparent C accumulation in the late Holocene (last 2000 years) from northern (n=122; ref. (5)) and tropical (n=7; ref. (49)) peatlands (data summarized in table S10). We used the late Holocene rates because longer time-records are characterized by variability caused by non-climatic factors and we were mainly interested in establishing relationships between C accumulation and present climate. There was no significant difference in the mean (or median) C accumulation of minerotrophic and ombrotrophic peatlands (ANOVA, Mann-Whitney test; $p>0.05$); nor were there a difference in the slope of the relationships between MAAT and C accumulation of minerotrophic and ombrotrophic peatlands (ANCOVA; $p>0.05$). There was insufficient data to estimate whether there was a difference in C accumulation between non-permafrost and permafrost peatlands. Thus, for the subsequent C accumulation analyses, all peatland types were grouped together. There was a significant linear relationship between C accumulation and MAAT (R2=0.27; $p<0.01$; AIC=29240; Fig. S9a). However, such a linear model unrealistically predicted negative C accumulation at MAAT below -18°C, where peatlands are known to exist. Based on our data points, the observed climate envelope of peatland sites has MAATs from -20° to +27° C. The best non-linear model fit is achieved with a logistic model (S-shaped curve) that is able to model growth with saturation at both high and low temperatures(50) (AIC=28822; Fig. S9b). This model predicted a C accumulation of ca. 5 g C m^{-2} yr^{-1} at MAAT -18°C, where the linear model becomes negative. Modelled C accumulation approaches zero at -40° C, but never becomes negative. At very high temperatures, it saturates below 64 g C m^{-2} yr^{-1}. The predicted long-term mean C accumulation scaled for the extratropical northern hemisphere under present baseline climate is 34 g C m^{-2} yr^{-1}. This model represents the best fit to the available data, but it is important to point out that the relationship is tenuous, as each site has been affected by multiple factors over the past 2000 years.

To assess the validity of our approach for estimating CO_2-C as a residual from long-term paleo-data, we compare it to data from the literature. For non-permafrost peatlands we estimate residual CO_2-C fluxes of 36 and 61 g C m^{-2} yr^{-1}, respectively for ombrotrophic and minerotrophic peatlands while the numbers for permafrost peatlands are 27 and 34 g C m^{-2} yr^{-1} (based on MAAT of +6° C and -2° C, respectively). These numbers are similar to typical values reported from the literature (summarized in Dataset S1). We note that there is a potential bias in our estimates since the long-term C accumulation rate accounts for how natural fire dynamics have influenced peatland C, while this is not included to short-term GHG flux measurements.

S1.5.1.2 Net loss of C and N after permafrost thaw from chronosequences

The net C budget following permafrost thaw was based on chronosequence studies of post-thaw permafrost peatlands (39, 41). Old permafrost C is lost following thaw, while increased ecosystem productivity in the young thermokarst (post-collapse) means that the surface peat is gaining C. In the early thaw stages the loss of old C is more rapid than the gain of new C, but there are differences between peatland types and sites. A compilation of five permafrost peatland thaw chronosequences (three epigenetic, two syngenetic, compiled in figure S7 in Turetsky et al. (2020) (41), show that epigenetic permafrost peatlands loose less deep C than syngeneic peatlands. There is an average net C loss of 19% of the total initial C storage after 100 yrs across all five chronosequences. Jones et al. (2017) (39) show that the loss of old permafrost C can be estimated as a function of pre-thaw C storage, but these results are based on syngenetic peatlands only. Assuming that the difference between epigenetic and syngenetic sites scale equally across sites with different initial peat C storage, we adapted the curves describing loss as a function of pre-thaw peat C storage from Jones et al to the mean of the five chronosequences. Table S7 of the supplement shows this adapted relationship between pre-thaw C and fraction of reminaing post-thaw C as a function of time extracted from figure 6 in Jones et al (39). The C loss during the first 100 years after thaw was estimated from the peatland C stock maps using the simplified equation $y= 1.1451x^{-0.0771}$ where y is the fraction of pre-thaw C that is lost in 100 years after thaw and x is the storage of pre-thaw C in kg C m^{-2} ($R^2 = 0.93$, from 100 years in Tab. S12). We scaled the changes in N pools from the C pools based on typical C:N ratios of permafrost peatlands and non-permafrost peatlands in tundra regions and boreal regions (table S6). We assumed these net peat C and N losses occur during the active layer deepening and young thermokarst stages and attributed the residual between our GHGs budgets (Tab. S4) and the predicted thaw-loss from chronosequences to lateral losses as dissolved or particulate organic matter in fluvial/aquatic systems. We considered a fluvial pathway more likely than GHG losses. To reconcile the C balance, annual C losses of several kg C yr^{-1} are needed (see ref. (45)) and our meta-analyses of GHG fluxes reveal no records of post-thaw C fluxes of that magnitude. However, we note that the observational network for monitoring thawing permafrost peatlands is very sparse, and rapid thaw-pulses of C could occur unobserved (41). Our approach here differed from that of ref. (41), who assumed that all the post-thaw losses were gaseous (CO_2 or CH_4). In our study, CO_2-C flux for the short-term post thaw stages were based on literature syntheses, but the mature collapse scar peatland and stable post-thaw peatlands were based on C accumulation modelled from projected future stabilized MAAT.

S1.5.1.3 Estimates of CH_4 fluxes

All data for estimated CH_4 fluxes were from a recent synthesis of year-round CH_4 fluxes in northern wetlands (51). We used only sites with organic soils (equals peatlands). We separated non-permafrost, permafrost and post-thaw sites. We further distinguished the minerotrophic peatlands (Swamp, Marsh and fen classes, following the Canadian wetland classification system) from ombrotrophic peatlands (bogs).

We tested if minerotrophic and ombrotrophic sites had significantly different median, or mean, CH_4 (Mann-Whitney test, ANOVA; $p<0.05$). For both non-permafrost and permafrost peatlands, there were significantly higher median/mean fluxes from minerotrophic peatlands. The Post-thaw landforms did not show significant differences between ombrotrophic and minerotrophic peatlands and no further distinction was made in the modelling. The annual CH_4 flux was not correlated to MAAT or MAP in non-permafrost, permafrost or post-thaw sites (Pearson linear correlation, $R^2 <0.1$; $p>0.05$) so no attempt was made to model CH_4 flux as a function of climate.

S1.5.1.4 Estimates of N_2O fluxes

For minerotrophic and ombrotrophic permafrost-free peatlands, we used annual N_2O budgets from a synthesis of N_2O fluxes from northern soils (52). Annual/seasonal N_2O data from Arctic peatlands are sparse: to our knowledge, they are limited to a single site located in Western Russia with discontinuous permafrost (“Seida”, 67°03’N, 62°57’E, 100m a.s.l.). We used published N_2O flux data from this site (53, 54) as N_2O emission estimates for minerotrophic and ombrotrophic (bare and vegetated) permafrost peatlands (Dataset S1). During the initial stage of gradual deepening of the active layer we modelled an increase in post-thaw N_2O emissions caused by N_2O production from N forms released with peat decomposition, and to a smaller extent, release of trapped N_2O (36). Data on N_2O (36) and CO_2 (46) fluxes from peat mesocosms during simulated permafrost thaw were used to develop a scaling ratio of N versus C release. First, we determined the theoretical amount of mobilized N, based on the C:N ratio with C lost as CO_2. From this theoretical amount of N (including all N forms such as N stored in the microbial biomass and N lost as leaching or N_2) we determined the percentage of N_2O -N lost based on measured N_2O fluxes. We used the obtained N_2O -N loss values of 7.38%

(bare peat) and 0.25% (vegetated peat) to calculate post-thaw N_2O fluxes relative to the estimated CO_2 fluxes. Bare peat is conservatively assumed to cover 3% of the peatland surface (55).

S1.5.2 Model of permafrost fraction in peatlands

The model of permafrost fraction in peatlands was derived using the method developed in ref. (56), where a relationship between permafrost fractional coverage and MAAT was fitted, by minimizing root mean squared error. For this study, the relationship was re-fitted using the fraction of permafrost in peatlands rather than the overall landscape fraction. Because the upper layers of peat often insulate the ground in summer and keep the soil cool the permafrost fraction in peatlands is usually higher than the overall landscape permafrost fraction because. The relationship between MAAT and peatland permafrost extent was represented by an error function with two parameters (μ and σ), based on ref. (57). For this study, the peatland maps did not reach 100% permafrost coverage even at very low MAAT. To model this, we introduced an additional overall scaling, f_{max}, representing the maximum permafrost coverage. This is consistent with small fractions of non-permafrost peatlands occurring also in very cold climates, usually caused by proximity to streams or lakes (37) (58). This gives the equation used as:

$$Permafrostfraction = 0.5 f_{max} ERFC\left(\frac{MAAT + \mu}{\sqrt{2\sigma^2}}\right)$$

where ERFC is the complementary error function (using the *pracma* R package).

As in ref (56), the curve was re-fitted using 'maximum' and 'minimum' permafrost fraction to give upper and lower estimates of permafrost fraction, as well as a central estimate. The maximum and minimum extents were derived from the highest and lowest per-pixel estimates of permafrost fraction in the national polygon maps and SoilGrids, respectively (with no MAAT correction applied to SoilGrids which allow larger spread of potential ecosystem-protected permafrost). Thus, three different parameter values (for central, upper and lower curves, respectively) were fitted for μ (1.95, 0.7 and 3.1), σ (7.35, 6.1 and 4.5) and f_{max} (0.92, 0.96 and 0.86). The μ values were smaller than in ref. (56) indicating that, indeed, peatlands allow permafrost to persist in warmer climates than is typical for landscapes dominated by mineral soils.

The air temperature map for present day and future scenarios was as in ref. (56). We assumed that mapped permafrost extent at +0.5ºC global warming (relative to preindustrial levels) was in quasi-equilibrium with the climate of the 1960 to 1990 period, which we also consider to be representative for the mapped permafrost extent in the maps of peatland permafrost fraction in ca. year 1990-2000 (approximate age of the map input data, which allows a lag time in peatland permafrost extent response to climate of several decades, consistent with ref. (38)). Global warming stabilization scenarios at 0.5ºC intervals up to a maximum of 6ºC (representing a very high-end RCP 8.5 scenario) were used for the future projections.

S1.5.3 Modelling radiative forcing

The projected GHG budgets, including CO_2, CH_4, and N_2O fluxes, from the spatial model were used to calculate the future radiative forcing effect. A range of GHG flux scenarios (available in appendix Dataset S3) were exported from the spatial model and used as input in a radiative forcing model (59) with additional parameterization for N_2O and modifications to atmospheric CO_2 lifetimes (60). Separate GHG flux scenarios were calculated for stabilized permafrost conditions at 0.5 degree increments from 0° to +6° C global warming stabilization (background concentrations were stable anthropogenic present day emissions). Separate runs were also done for fluxes resulting from the transient thaw scenarios for each incremental warming (Fig. 2, Dataset S1). The net radiative effect of the transient thaw is calculated as the different between stable scenarios and the transient scenarios. To compare the magnitude of permafrost-peatland thaw emissions to anthropogenic emissions, radiative forcing from anthropogenic emissions together with peatland thaw emissions were compared to anthropogenic emissions alone. These emissions scenarios were retrieved from Climate Scoreboard (61) and are computed using the C-ROADS climate policy model (62). For these calculations we assume that +0.5°C global warming is consistent with peatland fluxes in 1990-2000 (assuming decadal lags in thaw response relative to the 1960-1990 climate normal) and that +1°C warming is consistent with present day.

S2: Maps of peatland and permafrost extent and assessments against local scale data

For the northern peatland region there are two, fully independent, sources of soil maps with substantially higher spatial resolution than the global maps in WISE30sec: (i) a collection of harmonized national soil survey maps created by soil scientists from field data, topographic maps

aerial photographs and satellite remote sensing data (14) (further adapted for this study, see methods) and (ii) the SoilGrids250 m dataset (13), a digital soil map created from a combination of environmental variables and soil profile data using machine learning algorithms. These maps are based on entirely different input data, where made using different methods and at different times by different soil science experts. Because of this, we consider them to be independent and partially complementary, also acknowledging that combining the two maps may yield more robust scaling of peatland extent and permafrost extent in peatlands. There is not a complete overlap in these maps (Fig. S1). In SoilGrids, 95% of the northern extratropical peatland extent occurs within the region that is covered by the national soil maps

There is a remarkable consistency in total mapped peatland extent between SoilGrids and the regional polygon maps (table S1). However, there are differences in the spatial distribution of peatlands and very substantial difference in the areas of mapped permafrost extent (Fig. S1). Because we are discussion soil maps we will from now on use USDA Soil taxonomy terminology (63). Here, non-permafrost organic soils are called Histosol and permafrost affected organic soils are called Histels. In the national soil maps, the combined cover of Histels and Histosols in the northern permafrost region (as defined by ref (64)) is 2.1 M km2, similar to the extent of Histels in SoilGrids. The maps thus agree on the sum of peatland extent across the full extratropical northern domain and within the permafrost region; the differences in Histel cover can thus be caused either by different spatial distribution patterns within these regions or in differences in the extent to which permafrost affects peatlands in the same location.

The extent of Histels in SoilGrids suggests that there may be up to 1 M km2 of permafrost peatlands not accounted for in previous studies of the permafrost C stock (such as refs. (10, 65). The Histels in SoilGrids are largely mapped in known permafrost regions; 90% of the Histels in SoilGrids are mapped within the extent of the Brown et al. circumpolar permafrost map (64) and 97% within the permafrost extent of another map based on global temperatures and topography (57). Permafrost in peatlands is known to be less sensitive to thaw than mineral soil permafrost (due to the insulating properties of peat (66)). The mapped extent in SoilGrids is thus not entirely unrealistic, but certainly warrants further investigation and ground-truthing against local scale datasets. Ref. (67) reviewed the extent of peatlands within the Siberian Yedoma region and found limited evidence of peat formation there. On the other hand, ref. (68) performed a meta-analyses of local vs regional soil maps and found evidence of underestimation of permafrost peatlands in the regional soil maps in e.g. Arctic Canada.

To assess which maps are more accurate, we evaluated them against local scale observations of peatland and permafrost extent, including the >7000 peat cores (point-to-pixel comparisons) in our database as well as high-resolution and extensively ground-truthed local scale maps which span over several pixels or polygons in the maps. In general, SoilGrida maps a more dispersed peatland distribution, while the national soil maps map denser peat coverage in core regions known for extensive peat formation (e.g. the West Siberian lowlands and the Hudson Bay lowlands) (Fig. S1). Overlaying the point observations of peat cores (n >7000) on the maps suggest that SoilGrids better captures sites that are located in areas of low peat density while the regional polygon soil maps seem to better describe regions of very high peatland density (Fig. S2). More than 2000 of the sampled peat cores are in locations that show zero peatland cover in the national soil maps (or WISE30sec). Such distribution patterns can partly be explained by the differences in scale between the datasets. Minimum mapping units in national soil surveys will have prevented mapping of small isolated peat complexes which can be mapped by SoilGrids250m. At the same time, SoilGrids is based on machine learning with no described validation and expert input of peatland distributions. This leads us to reason that the maps are partly complementary and that using a per-pixel mean of the products may be the most robust option.

For ground-truthing of permafrost extent (fraction of mapped organic soils that are Histels), only the C-stock peat cores (n=ca 700) have data on peatland type. We compared the permafrost extent in the maps against applying MAAT thresholds but find that both maps outperform any MAAT threshold applied. This analyses, and the scientific literature, shows that Histels may occur at MAAT >+1°C, but it is rare and usually occurs in areas where the permafrost persist as relict from previous colder climates (66, 69). Because we are interested in broad-scale patterns and less in isolated spots or relict permafrost, we apply a threshold where no Histels are mapped at MAAT >+1°C. This also addresses unrealistic distributions of Histels in some regions from SoilGrid250m. This threshold eliminates some problems with SoilGrids. For instance, there is a definite overestimation of permafrost extent in some regions within the original SoilGrids datasets. This is exemplified by extensive Histel occurrences in central Scandinavia which are known to be permafrost free.

Comparisons, like the ones described above, that uses individual point observations to evaluate coverage in larger aggregated polygons or pixels will always suffer from a scale miss-match (70). We

are also able to evaluate the maps against local/regional maps of high accuracy which span over several pixels or polygons (Table S3). The results from the map comparisons are not fully conclusive, but they do show that a mean between the two maps performs better than either of the individual maps in most cases. Taken together these ground-truthing analyses suggest that the maps are to some degree complementary and that a mean of the two maps is the most robust scaling available.

S3: Results on peat depth, stocks of OC and TN

The peat depth, and stocks of peat C and N are characterized by very high spatial variability (Fig. 1c, d). In all regions where there is a high data-density, there is also very substantial variability in both peat depth and peat C storage. The mean depth in all cores is 234±175 cm (range 40-2,000 cm; n=7,111), the mean peat C storage is 106±66 kg C m^{-2} (range 0.4-593 kg C m^{-2}; n= 782) and the mean peat N storage is 3.9±2.4 kg N m^{-2} (ranges from 0.3-11.5 kg N m^{-2}; n=105). Sites with data on OC and TN stocks are somewhat shallower. This is because the spatial distribution of peat cores is uneven, particularly for sites with OC and N data which are clustered in northern regions (Fig. 1c) with shallower peat. We used linear models to estimate peat C and N storage from peat depth for permafrost-free and permafrost peatlands, respectively. We developed a separate function to estimate peat C storage from peat depth for permafrost-free and permafrost peatlands, respectively (Fig. S3ab). The slope of the linear relationship between peat depth and peat C storage is significantly flatter for permafrost sites than for other peatland types (ANCOVA, $p<0.05$), which is the reason two separate models were used instead of joining the data. This log-transformed data using Major Axis (MA) linear models (31); intercepts set to zero; n= 334 and 446; slopes 0.85011 and 0.88263; R^2 0.77 and 0.57; both $p<0.0001$). The low R^2 for scaling of C storage from depth in permafrost peatland is likely caused by the natural variability in ground-ice affecting the C density of frozen peat.
Although the input data is limited and variable, we also developed separate function to estimate peat N storage from peat depth for permafrost-free and permafrost peatlands, respectively (Fig S3c,d; log-transformed data using Major Axis linear models; intercepts set to zero; n= 16 and 85; slopes 0.18162 and 0.31196; R^2 0.35 and 0.42; $p<0.05$ and $p<0.0001$). The large spread in this data is reflected in the broad uncertainty ranges of scaled peatland N stocks reported in the main text.

S4: Analyses of observed lateral losses from thawing peatlands in relation to projected chronosequence peat losses

In this sections we outline a brief analyses of how our projections of potential lateral peat C fluxes inferred from chronosequnces of permafrost peatland thaw compare to observations of lateral flux from the field. Datasets that allow quantification of DOC/POC losses from areas with a strong signal of thawing peat (but limited interference of other permafrost thaw signals) are rare. We have found two examples, one from Scotty Creek in Western Canada and one from the West Siberian Lowlands, and they show very different results.
The projected average net lateral losses for a +1° C (consistent with the warming signal we could detect with contemporary data) warming scenario from our spatial model is 11 kg C m^{-2} for the thawed peatland, spread over 100 years. We do not define how this C loss is distributed over the 100 year time-period, but we assume that losses are higher in the earlier period and should be in the range of 100-200 g C m^{-2} yr^{-1}.
The Scotty Creek catchment in the discontinuous permafrost zone of western Boreal Canada is a well studied site where data on peatland thaw over time, GHG flux data, chronosequence studies and lateral flux data is available. The data from this site does not support our projections as there is little sign of lateral peat C losses despite ongoing and past thaw. Since 1970, the areal coverage of permafrost peat plateaus has declined from 35 km2 to <20 km (total catchment area is 139 km2), a net areal loss of 15% (71). If all these peatlands lost C at the rates projected, it would result in a DOC export from the thawing peatlands alone at the catchment outlet of 15-30 g C m^{-2} yr^{-1}. But the DOC export from the catchment is observed to be only ~1.5 g C m^{-2} yr^{-1} (72). There is very limited POC export and also radiocarbon data indicating that most of the DOC is modern (72). We thus conclude that at Scotty Creek, there is presently no large lateral flux component that can explain the post-thaw losses. But there are also no large gaseous losses at thawing sites (47), while the chronosequence at this site shows substantial long-term losses which we are unable to constrain with the available flux data.
There is data to support large lateral losses from the West Siberian Lowlands (57). In a transect from no permafrost to continuous permafrost across small peatland dominated catchments. We do not have data to constrain the rate of peatland permafrost thaw in these catchments over recent decade. Even though this region is dominated by peatlands, it is unlikely that the average catchments in that area have lost as much as 15% of the full catchment area as peatlands alone; we assume that they were

lower or comparable to those at Scotty Creek along the southern fringes of permafrost. Serikova et al. (57) show a distinct peak in DOC fluxes in the sporadic permafrost zone, 15-20 g C m^{-2} yr^{-1}, declining to 6-8 g g C m^{-2} yr^{-1} in the continuous permafrost zone. We thus conclude, that in these streams the observational data supports lateral fluxes of the magnitude projected from the limited chronosequence data. There is also strong indirect support for substantial lateral looses of permafrost DOC from peatlands in the permafrost thaw-signal detected in the large Siberian Arctic rivers. Wild et al. (73) make use of time series data, including C isotopic data, to show that the DOC export attributable to permafrost thaw is by far the strongest in the Ob river (with limited permafrost cover but much thawing peatland area), but rather small in the Kolyma river (with much permafrost cover but very little peatland cover).

Supplemental figures

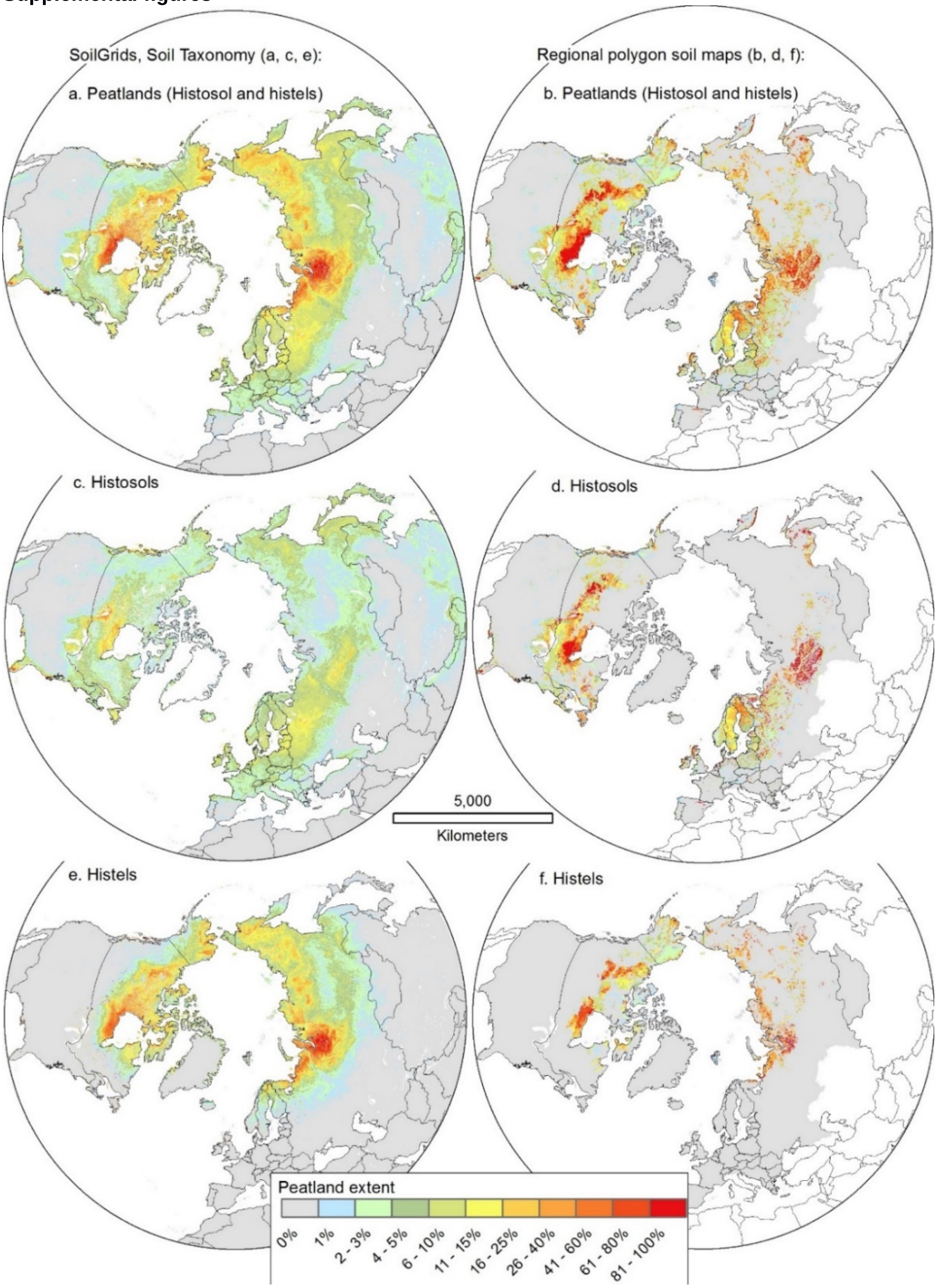

Figure S1. Maps of fractional peatland cover, subdivided into Histosols and histels, in in the SoilGrids dataset (panels a and b) and in the regional polygon soil maps (panels c and d). White land areas in c and d are not covered by the regional polygon soil maps. In the regional soil maps, the combined cover of Histels and Histotols in the northern permafrost region (as defined by Brown et al., 2002) is 2.1 M km2, similar to the extent of Histels in SoilGrids. In SoilGrids, 95% of the northern extratropical peatland extent occurs within the region that is covered by the regional soil maps.

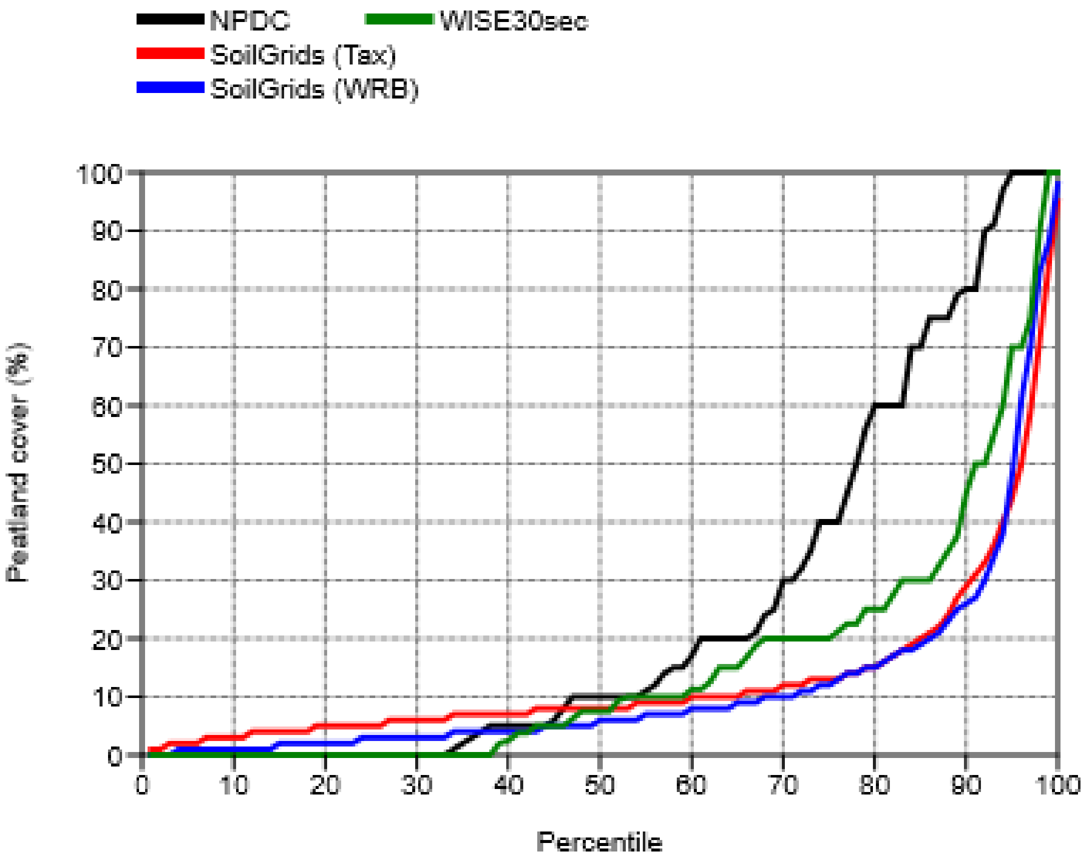

Figure S2. Percentiles of the peat core sites plotted against fraction of peatland cover in the different map products.

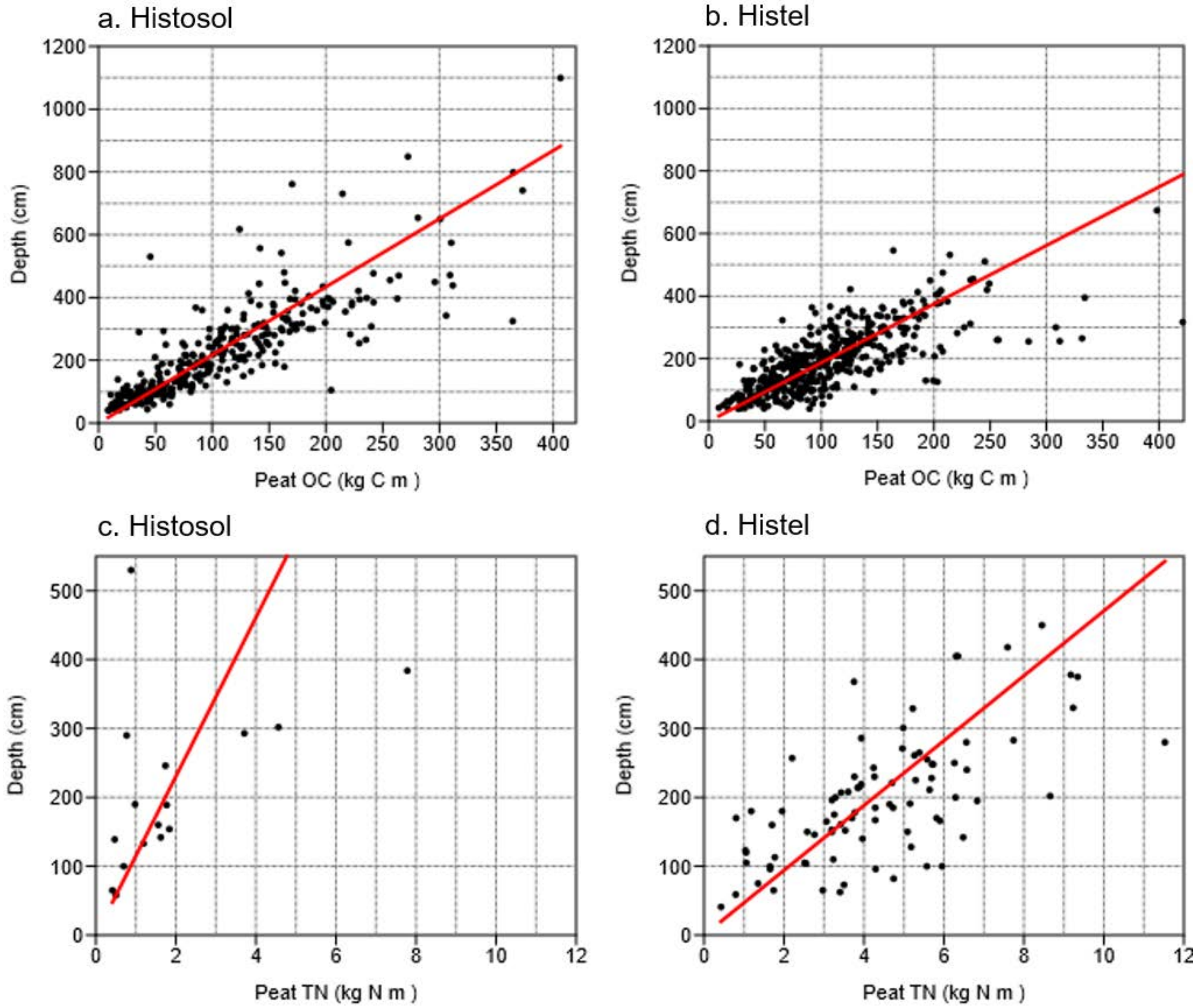

Figure S3. Peat depth in relation to organic C (OC) storage (a, b) and total N (TN) storage (c, d). Separate models were created for Histosols (a, c) and Histels (b, d). Figure shows MA linear models fitted to non-formatted data with intercept zero, for the calculations log-transformed data provided a better fit

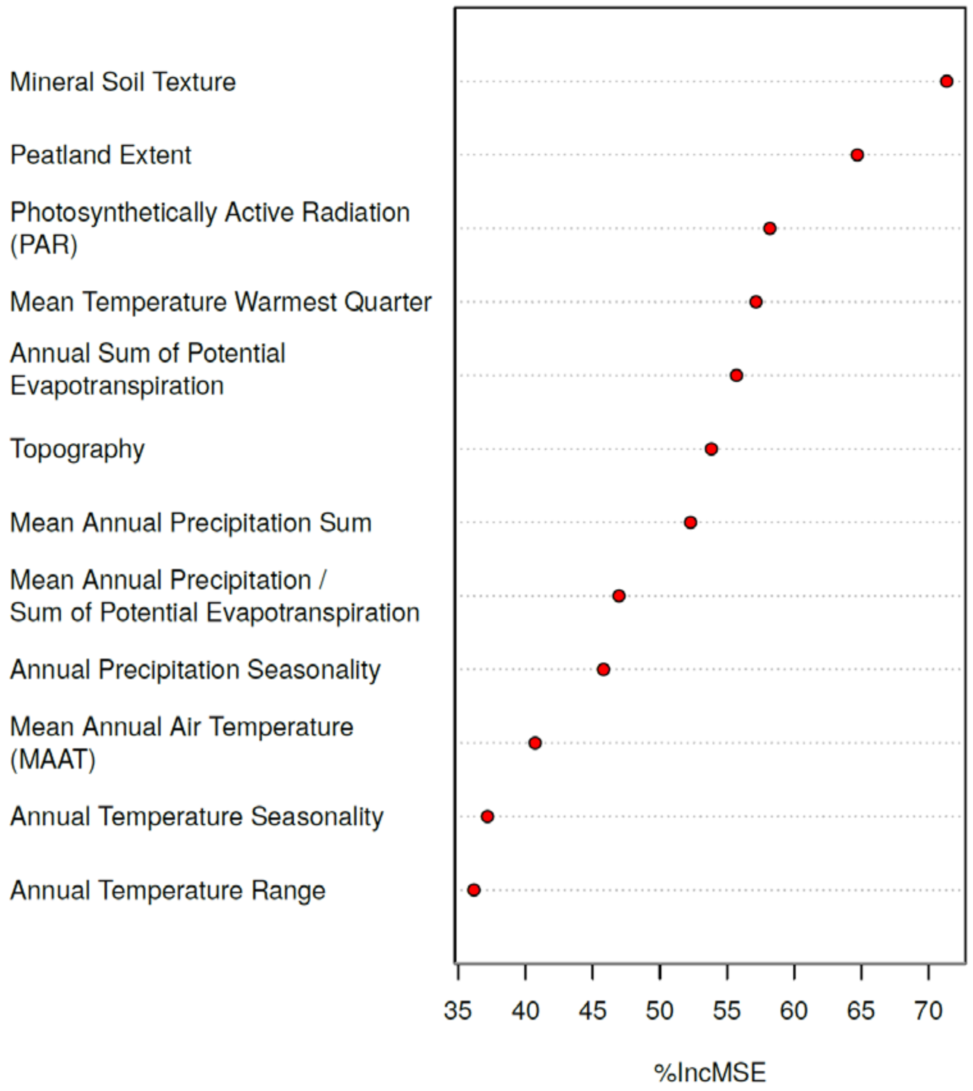

Figure S4. Variable importance plot for the RF model of peatland depth. Variable importance decreases from top to bottom and is measured in mean decrease in accuracy if the variable would be removed from the model (%IncMSE).

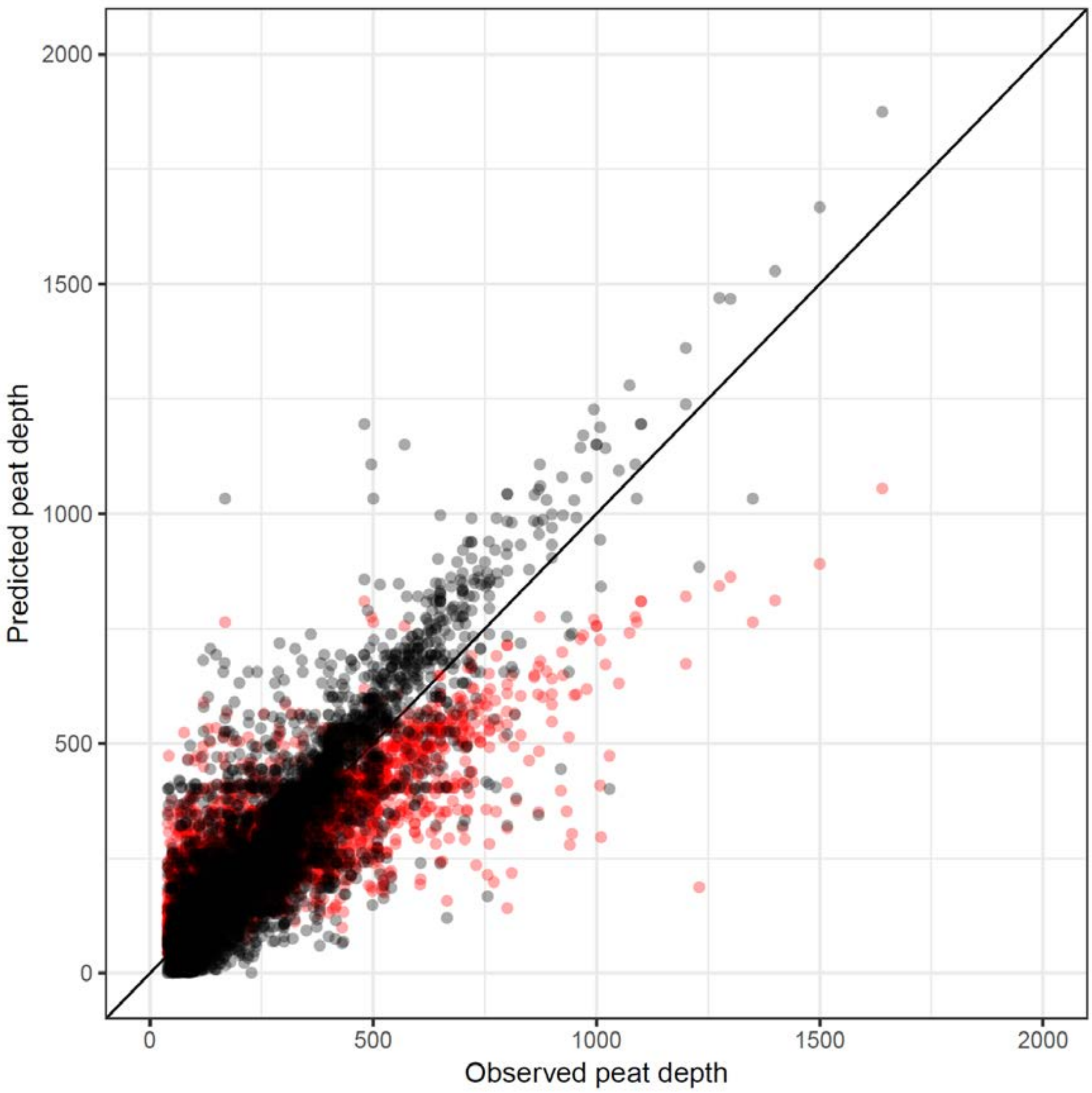

Fig S5: Comparison between the original Random Forest Machine Learning (red) showing a regression to mean effect that underestimates high values and the final, bias corrected, predicted values for peat depth (black).

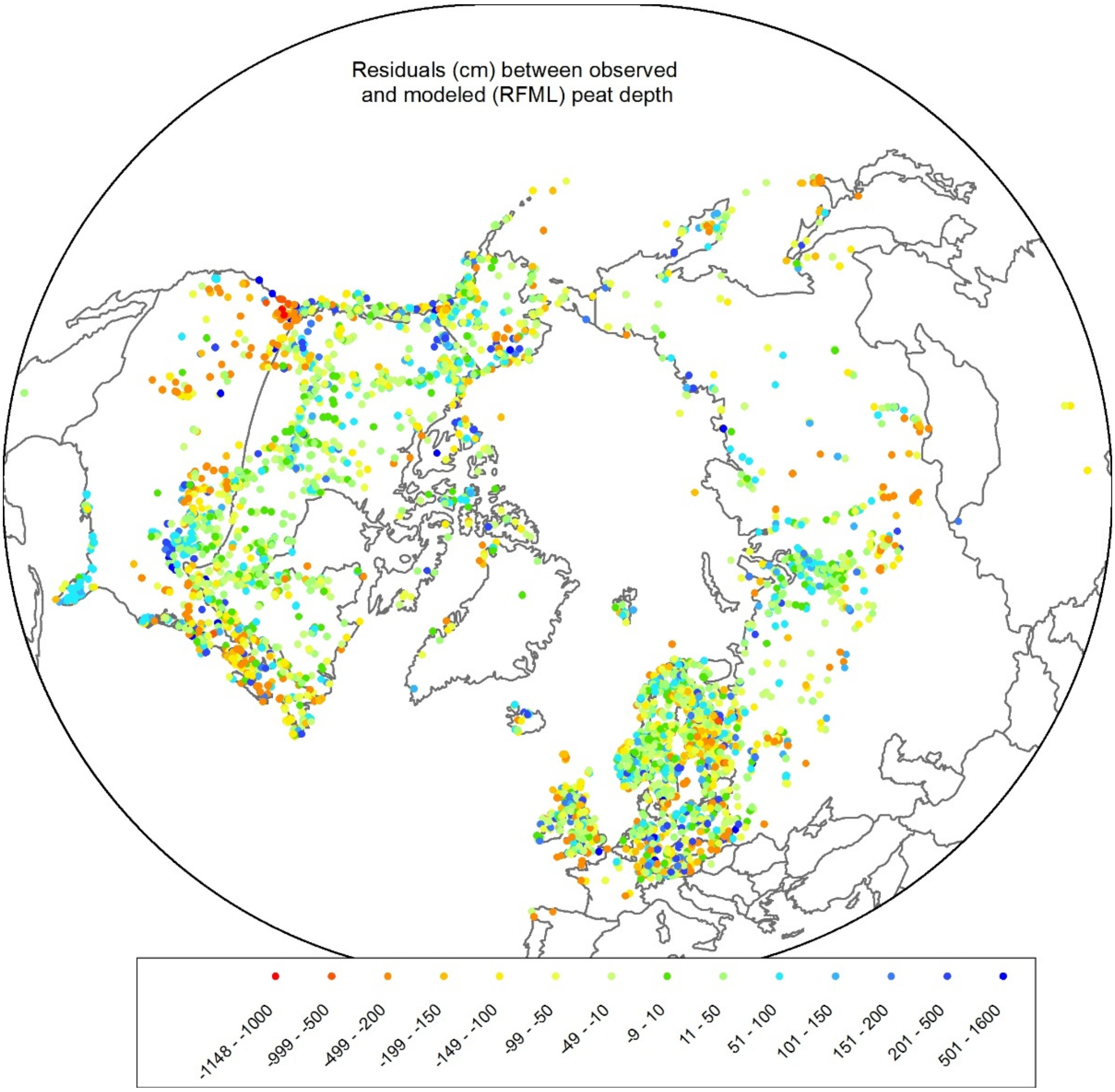

Figure S6. Map shows the residuals (cm) between observed and modeled peat depth. A positive residual means that the observed depth is greater than the modeled depth.

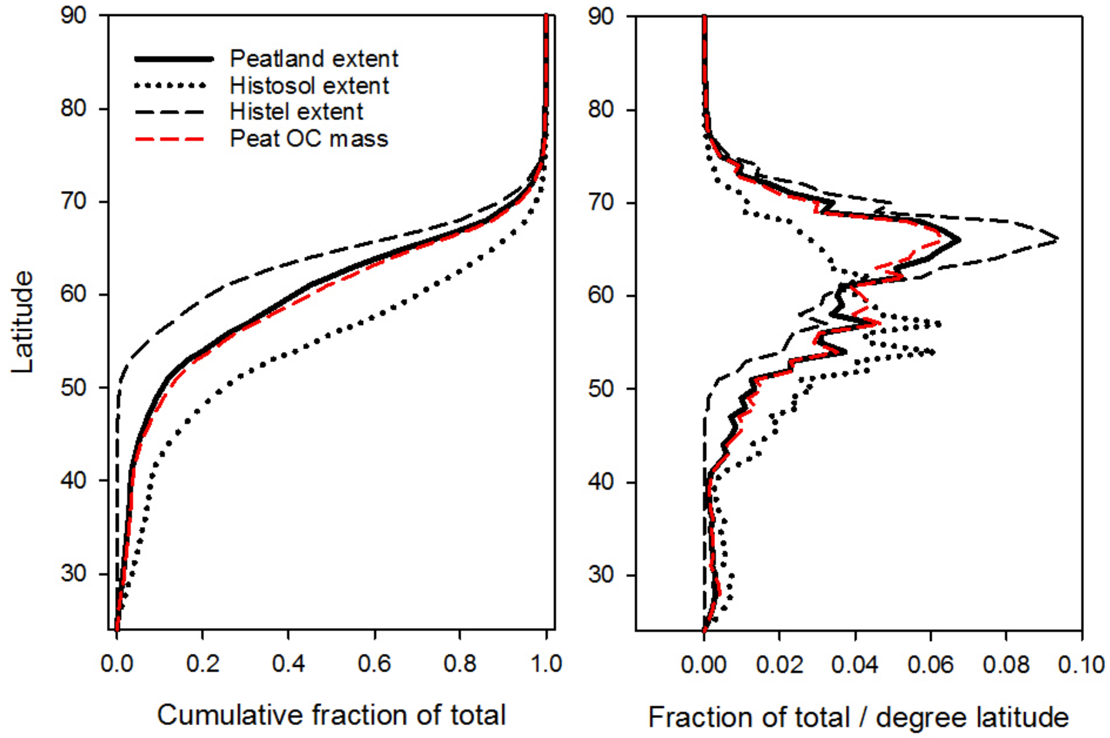

Figure S7. Latitudinal distribution of mapped peatland extent and C stocks. The first graph shows the cumulative fraction of peatland extent and OC mass with latitude north. The second graph shows the relative fraction per degree latitude.

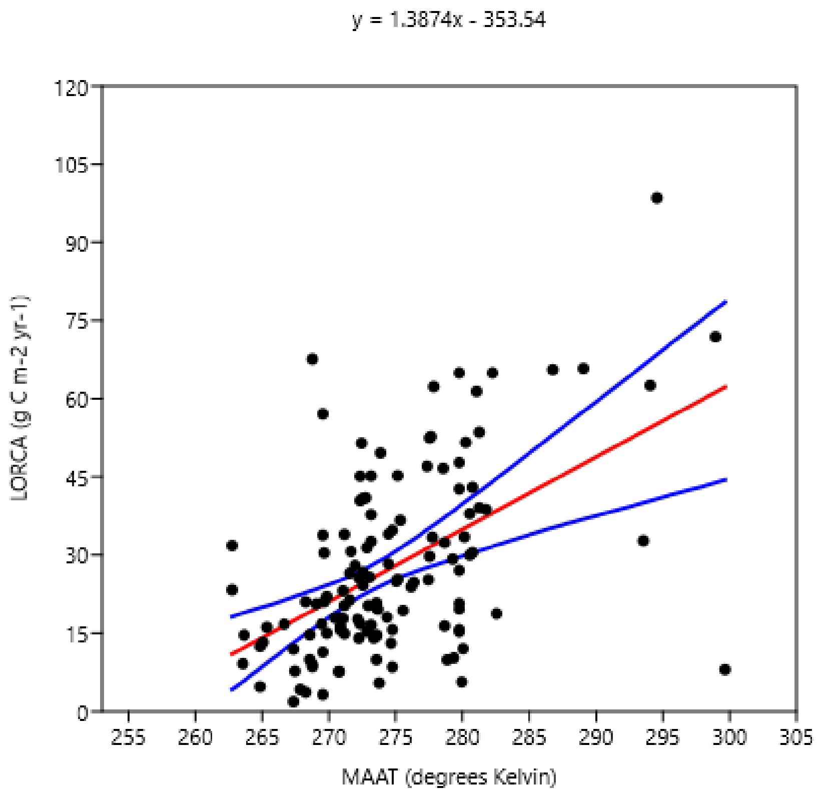

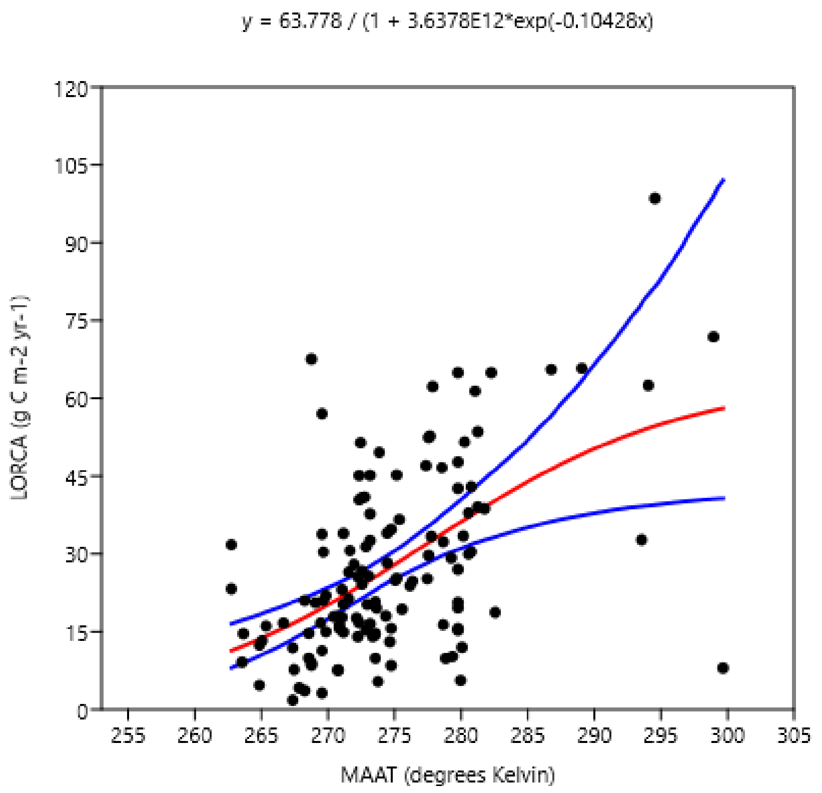

Fig S8. Linear (upper panel) and non-linear (lower panel) model fit between LORCA (g C m-2 yr-1) and MAAT (°K) with 95% confidence interval spread. Note that the temperature range (255 to 305 °K) on the x-axes is scaled to represent the observed climatic envelope of peatlands (MAAT from -20 to +27 °C). There is a particular lack of observations to support the model in very cold peat forming systems.

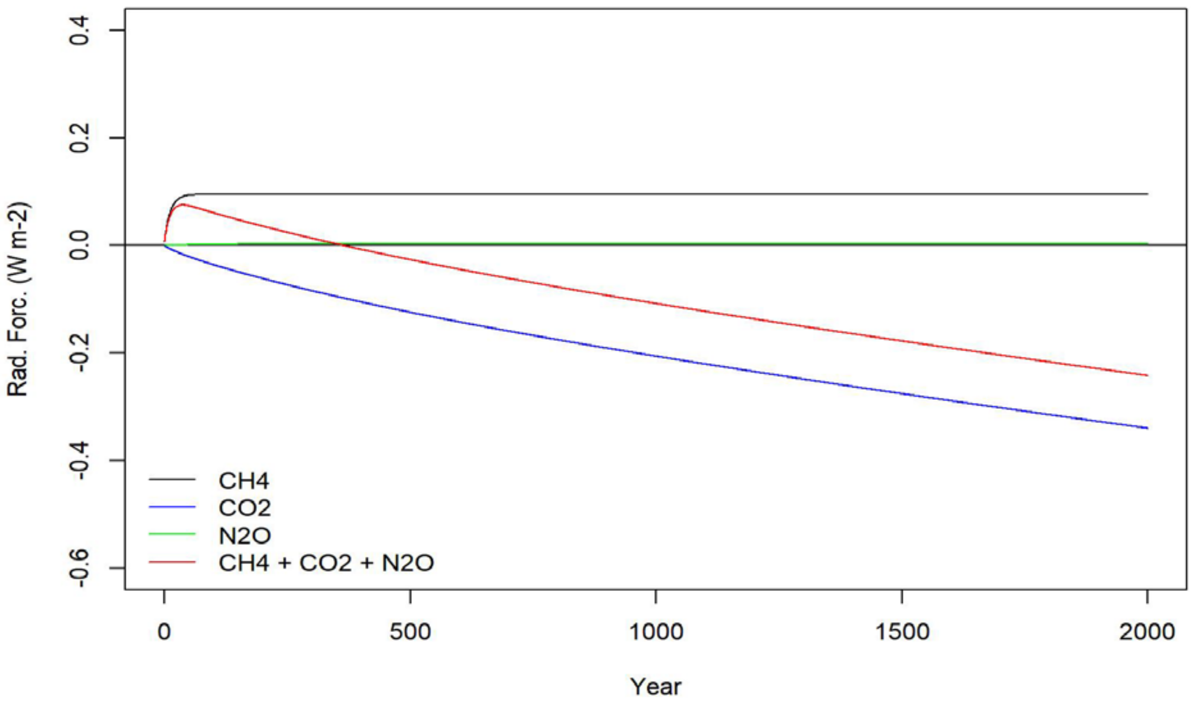

Figure S9. Radiative forcing of estimated present day emissions of CO_2, CH_4 and N_2O extended 2000 years into the futures, assuming constant emissions.

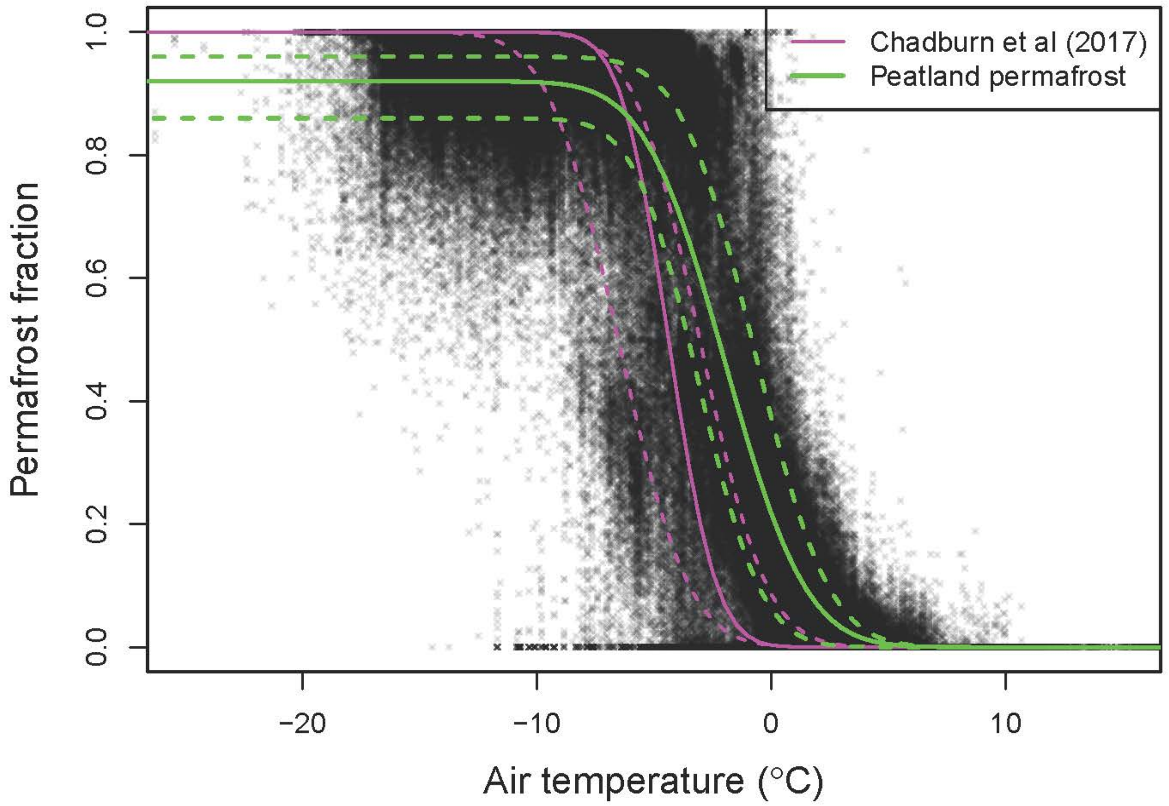

Figure S10. Curves showing the predicted permafrost fraction as a function of mean annual air temperature (MAAT). The original curve describing mineral soil permafrost (in pink) has been adapted to show predicted permafrost distribution in peatlands. The dotted lines of the green curves represent the spread between the lowest and highest mapped permafrost fractions in the national soil maps and SoilGrids products. Note that the peatland curve predicts ecosystem-protected permafrost occurring at MAAT >0°. The green curve includes a multiplicative factor which allows the curve to asymptote below 100% permafrost at cold air temperatures. This maximizes fit to the map data and implies that even in very cold places, some permafrost-free peatlands exist (due to e.g. local thermokarst or hydrological controls).

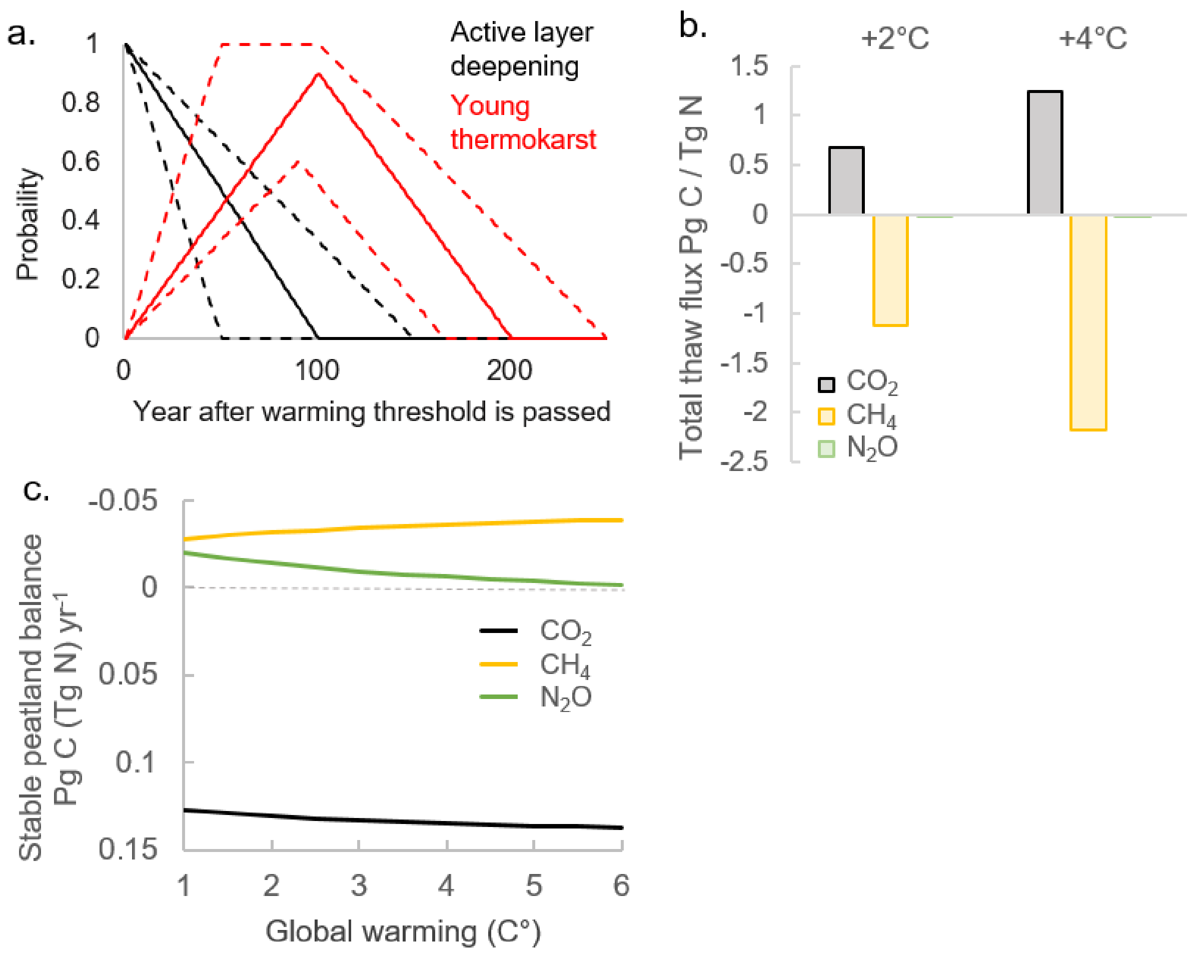

Figure S11. (a.) Time-dependent probability distributions of active layer warming/deepening (black lines) and young thermokarst stages (red lines) for peatlands in relation to time after the warming threshold that initiates thaw is passed. Dotted lines show minimum and maximum ranges which are accounted for in the flux uncertainty ranges. Note that 10% of sites do not go through the thermokarst stage. (b.) Figure summarizes the total net post-thaw losses of C and N as GHGs during the active layer deepening and young thermokarst stages, before the system enters the mature thermokarst phase. (c) Stabilized annual (post thaw) GHG balance for northern peatlands under different global warming. Negative numbers show a flux into the atmosphere (upwards in the figure).

Supplemental tables

Table S1. Table summarizing estimated peatland extent for various geographic regions based on regional polygon soil maps, ref (14) modified in this study), SoilGrids (13), WISE30sec (12) and published peat inventories based on national or regional statistics from tables (74–76). The National Inventories of peat have typically used a definition of peatland as having ≥30 cm surface peat while soil maps define organic soils/peatlands as having ≥40 cm surface peat. For the WISE30sec and the National Inventories only combined peatland cover is reported. For references not included in the main manuscript, see complementary list at the end of the document.

		Estimated peatland area ($\times10^6$ km^2)			
Extent:		National soil maps	SoilGrids	WISE30sec	National Inventories
Regional*	Histosol	2.24	1.31	-	-
	Histel	1.22	2.18	-	-
	Peatlands	3.46	3.49	2.61	3.2 to 3.46**
Northern	Histosol	-	1.46	-	-
	Histel	-	2.20	-	-
	Peatlands	-	3.66	2.67	3.2 to 3.7
World	Histosol	-	2.47	-	-
	Histel	-	2.20	-	-
	Peatlands	-	4.67	3.30	3.8 to 4.4

*Includes: Canada, Europe (except Italy), Mongolia, USA and Russia. ** Includes: Russia, USA, Canada, Fennoscandia, Mongolia, Kazaksthan. Greenland, Iceland and Svalbard.

Table S2. Table summarizing the environmental variables which were analyzed in relation to peatland depth, OC stocks and TN stocks in the individual peat cores as well as to the final maps of peat depth, peat extent and scaled peat OC stocks. For references not included in the main manuscript, see complementary list at the end of the document.

Variable group	Description, source of data	Hypothesis for influence on peat depth
Radiation	PAR: Photosynthetically Active Radiation summed during the growing season (April to October; period 1961-1990) (77–79).	+ High PAR increases plant productivity in peatlands (80).
Temperature	MAAT: Mean Annual Air temperature (period 1950-2000) (81).	+ High MAAT increases plant productivity in peatlands (- but may also increase peat decomposition rates and reduce permafrost protection of peat) (82).
	MTWQ: Mean Temperature of the years Warmest Quarter (period 1950-2000) (81).	+ High MTWQ increases plant productivity in peatlands (but may also increase peat decomposition rates and reduce permafrost protection of peat) (82).
	TempSeason: Annual Temperature Seasonality (Coefficient of Variation; period 1950-2000) (81).	+/- High TempSeason reflects warm productive summers and cold winters, but it is also correlated to continentality (82).
	TempAnnRange: Annual range of monthly mean temperature. (period 1950-2000) (81).	- High temperature ranges are associated to continental climates with limited peat accumulation (82).
Precipitation	MAP: Mean Annual Precipitation sum (period 1950-2000) (81).	+ High MAP favors waterlogging (which inhibits aerobic decay and increases peat formation (82).
	PrecipSeason: Annual Precipitation Seasonality (Coefficient of Variation; period 1950-2000) (81).	+/- May reflect both wet and dry conditions during the growing season
	PET: Annual sum of potential evapotranspiration (83).	- High PET limits waterlogging
	MAP/PET: The ratio of mean annual precipitation to annual sum of potential evapotranspiration.	+ High MAP/PET indicates precipitation excess which favours waterlogging
Mineral soil texture	Texture: Combined percentage of silt and clay (cohesion soils) mapped in the HWSD (17).	+ Fine textured cohesion soils reduces soil drainage favouring waterlogging
Topography	Topography: A topographic landform classification where rugged terrain has lower values than flat terrain or plains (84).	+ Low-lying and flat landforms are more prone to peat formation (82).
Peatland extent	Extent: Combined percentage coverage of organic soils. Source: this study	+ Internal controls of peatland hydrology and plant communities promotes continued peat formation

Table S3. Summary of estimated coverage of peatlands in local study areas compared to SoilGrids (SG) (13), national/regional harmonized soil survey maps (NM) (14) and the mean between the two map products (which is what was used in this paper).

			Histel extent (%)				Histolsol extent (%)				Organic soil extent (%)				Permafrost in peatlands (%)				Peat depth (cm)	
Lat.	Long.	Area	Local	SG	NM	Mean	Local	SG	NM	Mean	Local	SG	NM	Mean	Local	SG	Polygon	Mean	Local	RFML
68.34	19.06	Abisko, Scandinavia	1	3	0	2	5	2	15	9	6	5	15	10	11	60	0	30	55±50	77
72.47	101.90	Ary-Mas, N Siberia	2	12	14	13	0	1	0	1	2	13	14	14	100	92	100	96	133±40	161
71.30	-156.60	Barrow, N Alaska	1	10	20	15	0	2	0	1	1	12	20	16	100	83	100	92	100±100	113
68.75	161.48	Cherskii, NE Siberia	29	12	24	18	0	3	0	2	29	15	24	20	100	80	100	90	100±125	147
70.82	147.48	Kytalyk, N Siberia	0	12	22	17	0	2	0	1	0	14	22	18					-	184
72.45	126.35	LenaDelta, N Siberia	0	14	0	7	0	3	0	2	0	17	0	9					-	223
73.42	98.43	Logata, N Siberia	0	24	24	24	0	4	0	2	0	28	24	26					-	264
62.26	67.37	Rog 1, NW Russia	20	59	40	50	14	0	0	0	34	59	40	50	59	100	100	100	123±50	101
62.12	67.29	Rog 2, NW Russia	16	67	35	51	14	0	0	0	30	67	35	51	53	100	100	100	123±70	112
61.85	67.15	Rog 3, NW Russia	26	72	60	66	18	1	0	1	44	73	60	67	59	99	100	99	153±87	104
62.94	67.06	Seida, NW Russia	16	46	15	31	5	2	7	5	21	48	22	35	75	96	68	82	233±106	157
69.45	164.80	Shalaurova, NE Siberia	11	9	0	5	0	2	0	1	11	11	0	6	100	82			160±160	218
62.23	129.62	Spasskaya Pad, Central Siberia	8	10	0	5	2	1	0	1	10	11	0	6	80	91			99±78	263
62.92	-99.17	Tulemalu Lake, Central Canada	16	37	0	19	0	2	0	1	16	39	0	20	100	95			92±24	202
74.50	20.43	Zackenberg, E, Greenland	0.1	3	0	2	0	1	0	1	0	4	0	2	100	75			80±40	155
		Mean error:		16.3	7.2	11.8		-2.2	-2.4	-2.3		14.1	4.8	9.5		9.6	13.8	8.0		30.1
		RMSE:		24.4	16.1	18.8		7.1	7.3	6.9		19.7	12.8	14.1		28.9	26.5	24.3		70.9

Table S4. Summed of annual fluxes of greenhouse gases. Includes annual stable baseline fluxes from all peatlands at present day after temperature stabilization at +1.5 C above pre-industrial temperatures as well as transient annual fluxes from post-thaw peatlands, including active layer deepening and collapse scars. The standard error (SE) is calculated based on the coefficient of variation from the flux data used for scaling (table S4), for N20 it is the propagated error from N20 and C fluxes since N2O loss is scaled based on C loss.

	CO2-C (Pg C yr-1)	±SE	CH4-C (Pg C yr-1)	±SE	N20-N (Tg yr-1)	±SE
Present baseline flux (all peatlands)	0.105	0.018	-0.026	-0.002	-0.022	-0.005
Stabilized at +1.5 C (all peatlands)	0.113	0.019	-0.030	-0.002	-0.017	-0.004
Transient flux from Thawed peatlands:						
From active layer deepening	-0.038	-0.008	-0.001	0.000	0.002	0.001
From young collapse scars	0.016	0.005	-0.007	-0.001	0.001	0.000
From mature collapse scars	0.005	0.000	-0.006	-0.001	0.000	0.000

Table S5. Fractions of ombrotrophic and minerotrophic peatlands in different biomes. Based on spatial intersection of the Peatlands of Canada map (47) and a global biome map (48). The zone “other” includes Temperate, Oceanic, Mountain and Prairie regions.

	All peatlands		Permafrost peatlands		Non-permafrost peatlands	
Biome:	Minerotrophic	Ombrotrophic	Minerotrophic	Ombrotrophic	Minerotrophic	Ombrotrophic
Tundra	37%	63%	24%	76%	59%	41%
Boreal	31%	69%	8%	92%	38%	62%
Other	23%	77%	3%	97%	29%	71%
All Cananda	33%	67%	17%	83%	42%	58%

Table S6. Mass ratios of C/N for full peatland profiles as well as fraction of peatlands which have C:N ratios below 25 and 30 respectively. These fractions are used in the estimation of N_2O flux as a fraction of CO2 flux. For permafrost peatlands, separate numbers are calculated for the Tundra and Boreal biomes.

Peatland type	Mean	SD	Median	n	Fraction <25	Fraction <30
Non-permafrost, All	37	14	35	16	0.13	0.25
Permafrost, All	32	35	24	89	0.58	0.76
Permafrost, Boreal	37	41	27	45	0.38	0.60
Permafrost, Tundra	38	41	24	44	0.52	0.72

Table S7. The loss of old permafrost C can be estimated as a function of pre-thaw C stock. Table shows the relationship between pre-thaw C (initial permafrost peatland C stock) and fraction of pre-thaw C as a function of time. The fraction of C remaining at 100 yrs is based on a set of five chronosequences (2 syngenetic and 3 epigenetic peatland complexes) from ref (41) (Figure S7), the time dynamics and shifts with C stocks are based on figure 6 from ref. (39) , assuming that the dynamics scale linearly after including additional chronosequences sites.

	Initial permafrost peatland C stock (kg C m^{-2})				
yrs after thaw	88	78	68	56	37
	Fraction of pre-thaw C stock				
0	1.00	1.00	1.00	1.00	1.00
10	0.78	0.78	0.78	0.79	0.79
25	0.79	0.79	0.79	0.80	0.81
50	0.80	0.81	0.81	0.81	0.82
75	0.80	0.82	0.82	0.82	0.86
100	0.81	0.82	0.83	0.83	0.87
300	0.89	0.90	0.90	0.90	0.92
500	0.87	0.88	0.89	0.90	0.97
750	0.90	0.92	0.93	0.97	1.06
1000	0.92	0.95	0.97	1.00	1.12

Supplemental References

1. O. Hammer, D. A. T. Harper, P. D. Ryan, PAST: Paleontological Statistics Software Package for Education and Data Analysis. 9.

2. R Core Team, R: A Language and Environment for Statistical Computing. *R Found. Stat. Comput. Vienna Austria* (2016).

3. E. Gorham, C. Lehman, A. Dyke, D. Clymo, J. Janssens, Long-term carbon sequestration in North American peatlands. *Quat. Sci. Rev.* **58**, 77–82 (2012).

4. G. Hugelius, *et al.*, A new data set for estimating organic carbon storage to 3 m depth in soils of the northern circumpolar permafrost region. *Earth Syst. Sci. Data* **5**, 393–402 (2013).

5. J. Loisel, *et al.*, A database and synthesis of northern peatland soil properties and Holocene carbon and nitrogen accumulation. *The Holocene* **24**, 1028–1042 (2014).

6. G. M. MacDonald, *et al.*, Rapid Early Development of Circumarctic Peatlands and Atmospheric CH4 and CO2 Variations. *Science* **314**, 285–288 (2006).

7. M. S. Packalen, S. A. Finkelstein, J. W. McLaughlin, Carbon storage and potential methane production in the Hudson Bay Lowlands since mid-Holocene peat initiation. *Nat. Commun.* **5**, 4078 (2014).

8. C. C. Treat, *et al.*, Effects of permafrost aggradation on peat properties as determined from a pan-Arctic synthesis of plant macrofossils: Permafrost Effects on Peat Properties. *J. Geophys. Res. Biogeosciences* **121**, 78–94 (2016).

9. G. Hugelius, *et al.*, A new data set for estimating organic carbon storage to 3 m depth in soils of the northern circumpolar permafrost region. *Earth Syst. Sci. Data* **5**, 393–402 (2013).

10. G. Hugelius, *et al.*, Estimated stocks of circumpolar permafrost carbon with quantified uncertainty ranges and identified data gaps. *Biogeosciences* **11**, 6573–6593 (2014).

11. J. Loisel, *et al.*, Insights and issues with estimating northern peatland carbon stocks and fluxes since the Last Glacial Maximum. *Earth-Sci. Rev.* **165**, 59–80 (2017).

12. N. H. Batjes, Harmonized soil property values for broad-scale modelling (WISE30sec) with estimates of global soil carbon stocks. *Geoderma* **269**, 61–68 (2016).

13. T. Hengl, *et al.*, SoilGrids250m: Global gridded soil information based on machine learning. *PLOS ONE* **12**, e0169748 (2017).

14. Cryosol Working Group, Northern and Mid Latitudes Soil Database, Version 1. National Soil Database. *Res. Branch Agric. Agri-Food Can. Ott. Can.*

15. B. Lehner, P. Döll, Development and validation of a global database of lakes, reservoirs and wetlands. *J. Hydrol.* **296**, 1–22 (2004).

16. J. Xu, P. J. Morris, J. Liu, J. Holden, PEATMAP: Refining estimates of global peatland distribution based on a meta-analysis. *CATENA* **160**, 134–140 (2018).

17. FAO, IIASA, ISRIC, ISSCAS, JRC, Harmonized World Soil Database (version 1.2). *FAO IIASA Rome Italy Laxenburg Austria* (2012).

18. G. Hugelius, *et al.*, The Northern Circumpolar Soil Carbon Database: spatially distributed datasets of soil coverage and soil carbon storage in the northern permafrost regions. 11 (2013).

19. D. J. Lary, A. H. Alavi, A. H. Gandomi, A. L. Walker, Machine learning in geosciences and remote sensing. *Geosci. Front.* **7**, 3–10 (2016).

20. G. Forkuor, O. K. L. Hounkpatin, G. Welp, M. Thiel, High Resolution Mapping of Soil Properties Using Remote Sensing Variables in South-Western Burkina Faso: A Comparison of Machine Learning and Multiple Linear Regression Models. *PLOS ONE* **12**, e0170478 (2017).

21. S. Lamichhane, L. Kumar, B. Wilson, Digital soil mapping algorithms and covariates for soil organic carbon mapping and their implications: A review. *Geoderma* **352**, 395–413 (2019).

22. J. Li, A. D. Heap, A. Potter, J. J. Daniell, Application of machine learning methods to spatial interpolation of environmental variables. *Environ. Model. Softw.* **26**, 1647–1659 (2011).

23. Rudiyanto, *et al.*, Digital mapping for cost-effective and accurate prediction of the depth and carbon stocks in Indonesian peatlands. *Geoderma* **272**, 20–31 (2016).

24. M. B. Siewert, High-resolution digital mapping of soil organic carbon in permafrost terrain using machine learning: a case study in a sub-Arctic peatland environment. *Biogeosciences* **15**, 1663–1682 (2018).

25. L. Breiman, Random Forests. *Mach. Learn.* **45**, 5–32 (2001).

26. A. B. McBratney, M. L. Mendonça Santos, B. Minasny, On digital soil mapping. *Geoderma* **117**, 3–52 (2003).

27. B. P. Malone, B. Minasny, A. B. McBratney, *Using R for Digital Soil Mapping* (Springer International Publishing, 2017) https:/doi.org/10.1007/978-3-319-44327-0 (March 25, 2020).

28. M. Kuhn, Building Predictive Models in *R* Using the **caret** Package. *J. Stat. Softw.* **28** (2008).

29. J. Song, Bias corrections for Random Forest in regression using residual rotation. *J. Korean Stat. Soc.* **44**, 321–326 (2015).

30. L. I. Lin, A concordance correlation coefficient to evaluate reproducibility. *Biometrics* **45**, 255–268 (1989).

31. D. I. Warton, I. J. Wright, D. S. Falster, M. Westoby, Bivariate line-fitting methods for allometry. *Biol. Rev.* **81**, 259 (2006).

32. D. Olefeldt, N. T. Roulet, Effects of permafrost and hydrology on the composition and transport of dissolved organic carbon in a subarctic peatland complex: DOC IN A SUBARCTIC PEATLAND COMPLEX. *J. Geophys. Res. Biogeosciences* **117** (2012).

33. R. F. Connon, W. L. Quinton, J. R. Craig, J. Hanisch, O. Sonnentag, The hydrology of interconnected bog complexes in discontinuous permafrost terrains: Hydrology of Interconnected Bogs in Discontinuous Permafrost. *Hydrol. Process.* **29**, 3831–3847 (2015).

34. K. M. Haynes, R. F. Connon, W. L. Quinton, Permafrost thaw induced drying of wetlands at Scotty Creek, NWT, Canada. *Environ. Res. Lett.* **13**, 114001 (2018).

35. H. J. Åkerman, M. Johansson, Thawing permafrost and thicker active layers in sub-arctic Sweden. *Permafr. Periglac. Process.* **19**, 279–292 (2008).

36. C. Voigt, *et al.*, Increased nitrous oxide emissions from Arctic peatlands after permafrost thaw. *Proc. Natl. Acad. Sci.* **114**, 6238–6243 (2017).

37. G. Hugelius, P. Kuhry, C. Tarnocai, T. Virtanen, Soil organic carbon pools in a periglacial landscape: a case study from the central Canadian Arctic. *Permafr. Periglac. Process.* **21**, 16–29 (2010).

38. G. Hugelius, *et al.*, High-resolution mapping of ecosystem carbon storage and potential effects of permafrost thaw in periglacial terrain, European Russian Arctic. *J. Geophys. Res.* **116**, G03024 (2011).

39. M. C. Jones, *et al.*, Rapid carbon loss and slow recovery following permafrost thaw in boreal peatlands. *Glob. Change Biol.* **23**, 1109–1127 (2017).

40. J. A. O'Donnell, *et al.*, The Effects of Permafrost Thaw on Soil Hydrologic, Thermal, and Carbon Dynamics in an Alaskan Peatland. *Ecosystems* **15**, 213–229 (2012).

41. M. R. Turetsky, *et al.*, Carbon release through abrupt permafrost thaw. *Nat. Geosci.* **13**, 138–143 (2020).

42. K. M. Walter Anthony, *et al.*, A shift of thermokarst lakes from carbon sources to sinks during the Holocene epoch. *Nature* **511**, 452–456 (2014).

43. G. Grosse, B. Jones, C. Arp, "8.21 Thermokarst Lakes, Drainage, and Drained Basins" in *Treatise on Geomorphology*, (Elsevier, 2013), pp. 325–353.

44. A. B. K. Sannel, P. Kuhry, Warming-induced destabilization of peat plateau/thermokarst lake complexes. *J. Geophys. Res.* **116**, G03035 (2011).

45. C. Estop-Aragonés, *et al.*, Limited release of previously-frozen C and increased new peat formation after thaw in permafrost peatlands. *Soil Biol. Biochem.* **118**, 115–129 (2018).

46. C. Voigt, *et al.*, Ecosystem carbon response of an Arctic peatland to simulated permafrost thaw. *Glob. Change Biol.* **25**, 1746–1764 (2019).

47. C. Tarnocai, I. M. Kettles, B. Lacelle, "Peatlands of Canada" (2011).

48. D. M. Olson, *et al.*, Terrestrial Ecoregions of the World: A New Map of Life on Earth. *BioScience* **51**, 933 (2001).

49. Z. Yu, J. Loisel, D. P. Brosseau, D. W. Beilman, S. J. Hunt, Global peatland dynamics since the Last Glacial Maximum: GLOBAL PEATLANDS SINCE THE LGM. *Geophys. Res. Lett.* **37**, n/a-n/a (2010).

50. D. Brown, P. Rothery, *Models in biology: mathematics, statistics and computing.* (John Wiley & Sons Ltd., 1993).

51. C. C. Treat, A. A. Bloom, M. E. Marushchak, Nongrowing season methane emissions-a significant component of annual emissions across northern ecosystems. *Glob. Change Biol.* **24**, 3331–3343 (2018).

52. M. Maljanen, *et al.*, Greenhouse gas balances of managed peatlands in the Nordic countries – present knowledge and gaps. *Biogeosciences* **7**, 2711–2738 (2010).

53. M. E. Marushchak, *et al.*, Hot spots for nitrous oxide emissions found in different types of permafrost peatlands: NITROUS OXIDE FLUXES FROM PERMAFROST PEATLANDS. *Glob. Change Biol.* **17**, 2601–2614 (2011).

54. C. Voigt, *et al.*, Warming of subarctic tundra increases emissions of all three important greenhouse gases - carbon dioxide, methane, and nitrous oxide. *Glob. Change Biol.* **23**, 3121–3138 (2017).

55. M. E. Repo, *et al.*, Large N2O emissions from cryoturbated peat soil in tundra. *Nat. Geosci.* **2**, 189–192 (2009).

56. S. E. Chadburn, *et al.*, An observation-based constraint on permafrost loss as a function of global warming. *Nat. Clim. Change* **7**, 340–344 (2017).

57. S. Gruber, Derivation and analysis of a high-resolution estimate of global permafrost zonation. *The Cryosphere* **6**, 221–233 (2012).

58. M. B. Siewert, *et al.*, Comparing carbon storage of Siberian tundra and taiga permafrost ecosystems at very high spatial resolution: ECOSYSTEM CARBON IN TAIGA AND TUNDRA. *J. Geophys. Res. Biogeosciences* **120**, 1973–1994 (2015).

59. S. Frolking, N. T. Roulet, Holocene radiative forcing impact of northern peatland carbon accumulation and methane emissions. *Glob. Change Biol.* **13**, 1079–1088 (2007).

60. R. Dommain, *et al.*, A radiative forcing analysis of tropical peatlands before and after their conversion to agricultural plantations. *Glob. Change Biol.* **24**, 5518–5533 (2018).

61. L. Siegel, Scoreboard Science and Data. *Clim. Interact.* (2017) (September 28, 2018).

62. J. D. Sterman, *et al.*, Management flight simulators to support climate negotiations. *Environ. Model. Softw.* **44**, 122–135 (2013).

63. Soil Survey Staff, Soil taxonomy: A basic system of soil classification for making and interpreting soil surveys. 2nd edition. *Atural Resour. Conserv. Serv. US Dep. Agric. Handb.* **436** (1999).

64. J. Brown, O. Ferrians, J. A. Heginbottom, E. Melnikov, Circum-Arctic Map of Permafrost and Ground-Ice Conditions, Version 2. [. *Boulder Colo. USA NSIDC Natl. Snow Ice Data Cent.* (2002).

65. C. Tarnocai, *et al.*, Soil organic carbon pools in the northern circumpolar permafrost region: SOIL ORGANIC CARBON POOLS. *Glob. Biogeochem. Cycles* **23**, n/a-n/a (2009).

66. Y. L. Shur, M. T. Jorgenson, Patterns of permafrost formation and degradation in relation to climate and ecosystems. *Permafr. Periglac. Process.* **18**, 7–19 (2007).

67. G. Hugelius, P. Kuhry, C. Tarnocai, Ideas and perspectives: Holocene thermokarst sediments of the Yedoma permafrost region do not increase the northern peatland carbon pool. *Biogeosciences* **13**, 2003–2010 (2016).

68. P. Kuhry, *et al.*, Characterisation of the Permafrost Carbon Pool: Permafrost Carbon. *Permafr. Periglac. Process.* **24**, 146–155 (2013).

69. A. B. K. Sannel, G. Hugelius, P. Jansson, P. Kuhry, Permafrost Warming in a Subarctic Peatland - Which Meteorological Controls are Most Important?: Factors Controlling Permafrost Warming in a Subarctic Peatland. *Permafr. Periglac. Process.* **27**, 177–188 (2016).

70. S. Openshaw, P. J. Taylor, A Million or so Correlation Coefficients: Three Experiments on the Modifiable Areal Unit Problem. *Stat. Appl. Spat. Sci.* **1**, 127–144 (1979).

71. L. Chasmer, C. Hopkinson, Threshold loss of discontinuous permafrost and landscape evolution. *Glob. Change Biol.* **23**, 2672–2686 (2017).

72. K. Burd, *et al.*, Seasonal shifts in export of DOC and nutrients from burned and unburned peatland-rich catchments, Northwest Territories, Canada. *Hydrol. Earth Syst. Sci.* **22**, 4455–4472 (2018).

73. B. Wild, *et al.*, Rivers across the Siberian Arctic unearth the patterns of carbon release from thawing permafrost. *Proc. Natl. Acad. Sci.* **116**, 10280–10285 (2019).

74. E. Gorham, Northern Peatlands: Role in the Carbon Cycle and Probable Responses to Climatic Warming. *Ecol. Appl.* **1**, 182–195 (1991).

75. E. Maltby, P. Immirzi, Carbon dynamics in peatlands and other wetland soils regional and global perspectives. *Chemosphere* **27**, 999–1023 (1993).

76. H. Joosten, D. Clarke, *Wise use of mires and peatlands: background and principles including a framework for decision-making* (International Peat Society ; International Mire Conservation Group, 2002).

77. F. G. Hall, *et al.*, ISLSCP Initiative II global data sets: Surface boundary conditions and atmospheric forcings for land-atmosphere studies. *J. Geophys. Res.* **111**, D22S01 (2006).

78. M. New, M. Hulme, P. Jones, Representing Twentieth-Century Space–Time Climate Variability. Part II: Development of 1901–96 Monthly Grids of Terrestrial Surface Climate. *J. Clim.* **13**, 22 (2000).

79. G. Papaioannou, N. Papanikolaou, D. Retalis, Relationships of photosynthetically active radiation and shortwave irradiance. 5.

80. J. Loisel, A. V. Gallego-Sala, Z. Yu, Global-scale pattern of peatland <i>Sphagnum</i> growth driven by photosynthetically active radiation and growing season length. *Biogeosciences* **9**, 2737–2746 (2012).

81. R. J. Hijmans, S. E. Cameron, J. L. Parra, P. G. Jones, A. Jarvis, Very high resolution interpolated climate surfaces for global land areas. *Int. J. Climatol.* **25**, 1965–1978 (2005).

82. E. Kivinen, P. Pakarinen, Geographical distribution of peat resources and major peatland complex types in the world. *Ann. Acad. Sci. Fenn. Ser. III Geol.-Geogr.* **132**, 1–29.

83. P. P. Hearn, “USGS Global GIS : global coverage database” (USGS).

84. J. Iwahashi, R. J. Pike, Automated classifications of topography from DEMs by an unsupervised nested-means algorithm and a three-part geometric signature. 32 (2007).
